# Caregiving-related experiences associated with depression severity and its symptomatology among caregivers of individuals with a severe mental disorder: an online cross-sectional study

**DOI:** 10.1007/s00406-022-01451-3

**Published:** 2022-06-30

**Authors:** Louis-Ferdinand Lespine, Anne-Lise Bohec, Jean-Michel Dorey, Céline Dubien Berbey, Charles Lourioux, Thierry D’amato, Marie-Odile Krebs, Isabelle Rouch, Romain Rey

**Affiliations:** 1 University Lyon 1, Villeurbanne, France; 2 Service Universitaire d’Addictologie de Lyon, Le Vinatier Hospital, Bron, France; 3 INSERM, U1028; CNRS, UMR5292; Lyon Neuroscience Research Center, Psychiatric Disorders: From Resistance to Response Team, Lyon, France; 4 INSERM U1028, CNRS, UMR 5292, Lyon Neuroscience Research Center, Eduwell, Lyon, France; 5 University of Paris, GHU-Paris, Sainte-Anne, C’JAAD, Hospitalo-Universitaire Department SHU, Inserm U1266, Institut de Psychiatrie (CNRS 3557), Paris, France; 6 Neurology Unit, CM2R, CHU de Saint Etienne, St-Priest-en-Jarez, France; 7 Schizophrenia Expert Center, Le Vinatier Hospital, Bron, France; 8 Fondation FondaMental, Créteil, France; 9 Lyon 2 University, Lyon, France; 10 Pôle PsyPA, Le Vinatier Hospital, Bron, France; 11 Clinical and Research Memory Center of Lyon, Villeurbanne, France; 12 INSERM, U1219, Bordeaux Population Health Center, University of Bordeaux, Bordeaux, France

**Keywords:** Caregiver, Depression, Burden, Mental illness, Psychiatry, Network analysis

## Abstract

Caring for a relative with a severe mental disorder puts family caregivers to a great risk of depression. While overall caregiving burden is a strong predictor of depression, the contribution of the various dimensions of burden to caregivers’ depression as well as their relationships with depressive symptoms has received little attention. 384 family caregivers completed a cross-sectional online survey including the Center for Epidemiological Studies Depression (CES-D) scale, the Zarit Burden Interview (ZBI), and the Brief Experience of Caregiving Inventory (BECI), measuring caregiving burden and experience. We estimated the structure of the relationships between caregiving experiences (i.e., ZBI and BECI subscales) and CES-D symptoms using a network approach. Negative Emotion/Consequences, (lack of) Positive Personal Experience, and Stigma/Effects on Family were the most connected caregiving dimensions to depression. To untangle the role of the Negative Emotion/Consequences component (by far the most central node in estimated networks), a secondary analysis incorporating its composing items was estimated. *Losing control over life*, *feeling strained around the relative* and *impaired self-perceived health* emerged as central nodes. Interestingly, these caregiving-related dimensions or experiences were differentially connected to depressive symptoms**.** We discuss how these findings might help future research and inform tailored psychoeducational interventions for family caregivers of people with a severe mental disorder.

**Supplementary Information:**

The online version contains supplementary material available at 10.1007/s00406-022-01451-3.

## Introduction

Family caregivers fulfill multiple roles in the care of subjects with a severe mental disorder (SMD) such as schizophrenia, bipolar disorder or major depressive disorder. In addition to providing help with activities of daily living, caregivers also provide emotional, social and financial support to individuals with SMD. In England, it was estimated that day-to-day care provided by caregivers to individuals with schizophrenic disorders saves governments and health care systems more than £1.24 billion a year [1]. In this regard, family caregivers can be seen as key actors and indispensable colleagues promoting recovery for people with SMD [2].

Due to their considerable care responsibilities, family caregivers are often unable to deal with their own individual or family needs. To compound this fact, family caregivers also face societal stigma, shame and prejudice. Caring for a person with a SMD is thus associated with deleterious consequences on mental and physical health [3, 4]. The negative psychological state experienced by informal caregivers, which arises from the various difficulties and stigma associated with caring for a relative with a SMD, has been defined as caregiver burden. Notably, caregivers of relatives with a SMD are at high risk for depression. Among caregivers of subjects with a schizophrenic or schizoaffective disorder, 42% were depressed [5]. Similarly, a comprehensive review examining psychiatric symptoms on caregivers of patients with bipolar disorders reported that 33–46% of caregivers met criteria for major depression [6].

Depression contributes to both distress and disability in caregivers while undermining their ability to carry out their essential supportive role toward their relative with a SMD [7]. Furthermore, depression is associated with various psychological and somatic problems [8], and a higher risk of suicide [9]. Considering the high rate of depression among caregivers, it is essential to address this issue not only through treatments after the depression onset, but also by developing interventions to prevent caregivers' depression. In this regard, it is of paramount importance to determine factors that contribute to depression in caregivers. While previous studies have investigated the risk factors of depression in caregivers, most of them related to caregivers of patients with cancer [10–14], brain injuries, neurodegenerative disorders or in pediatric settings [15–20]. Very few studies have explored the risk factors for depression in caregivers of patients with SMD (e.g., [21, 22]). Among caregivers of old adults with SMD, various predictors of depression have been reported such as low income, care recipient gender, poor health, and problems dealing with care recipient’s symptoms [22].

Remarkably, while overall caregiver burden has been identified as a strong predictor of caregiver depressive symptoms [21], the contribution of the various dimensions of burden to caregivers’ depression remains unknown. Indeed, caregiver burden is a multi-faceted construct reflecting various dimensions such as negative emotion, interpersonal relationship, time demand, patient’s dependence, and self-accusation and guilt [23]. Similarly, caregiving experience includes negative as well as positive aspects [24, 25]. To the best of our knowledge, no study has examined the contribution of the various dimensions of caregiving experience to the severity of depression and how they might relate to individual depressive symptoms in caregivers of people with a SMD. A better understanding of such complex inter-relationships can guide mental health professionals in identifying at-risk caregivers and providing them with specific interventions.

## Materials and methods

### Sample, design and setting

We conducted a cross-sectional online survey (https://framaforms.org) in France from April 6 to May 11 2020, i.e., during the first country-wide lockdown due to COVID-19 pandemic. Following a convenience non-probability sampling method, participants were recruited with online announcements on websites of mental health and mailing lists from caregivers associations, with no incentives. The inclusion criteria included (1) having a relative suffering from a mental illness, (2) being the primary caregiver, (3) speaking French, and (4) being at least 18 years of age. In line with French regulations on health research, no ethics committee approval was required because data collection was anonymous. This study is reported according to the ‘Checklist for Reporting Results of Internet E-Surveys’ (CHERRIES) statement [26, 27].

### Measures

The survey included socio-demographics, relative’s diagnosis, and personal and environmental conditions during lockdown (whose data were not analyzed in this study). Participants were also asked whether they had ever benefited from a psychoeducation program (‘yes’ or ‘no’).

*The Center for Epidemiological Studies Depression (CES-D) scale*. Depression symptoms were assessed using the 20-item CES-D scale [28, 29], a self-report measure where participants indicate how often over the past week various statements such as *I felt lonely* or *I felt sad* applied to them. Each item is rated on a 4-point Likert scale that ranges from 0 (rarely or none of the time) to 3 (most of the time), with total scores ranging from 0 to 60.

*The Zarit Burden Interview (ZBI)*. Caregiving burden was assessed using the 22-item version of the ZBI [30, 31], a self-report measure where individuals indicate how often statements such as *you feel that your relative asks for more help than he/she needs* or *you feel embarrassed about your relative’s behaviour* apply to them. Each item is rated on a 5-point Likert scale that ranges from 0 (never) to 4 (nearly always), total scores ranging from 0 to 88. The 22-item ZBI has demonstrated good reliability and validity across studies [32]. However, factor structures varied across studies, which may rely in part on differences in the composition of caregiver samples. Therefore, we used the structure reported by Tang et al. 2017 in a sample of caregivers of patients with schizophrenia [23]. Subscales (i.e., factors) include Negative Emotion/Consequences, Interpersonal Relationship, Time Demand, Patient’s Dependence, and Self-accusation/Guilt.

*The Brief Experience of Caregiving Inventory (BECI)*. Caregiving experience was further assessed using the BECI [24], a 19-item self-report measure where individuals indicate how often over the past month statements such as *I have thought about feeling unable to tell anyone of the illness* or *the illness is causing a family breakup* applied to them. Each item is rated on a 5-point Likert scale that ranges from 0 (never) to 4 (nearly always), with total scores ranging from 0 to 76. The validation process carried out on data collected in 626 carers of individuals with psychosis resulted in a 19-item, four-factor inventory with a good model fit, and displaying good reliability and validity [24]. Subscales (i.e., factors) include Difficult Behaviours, Positive Personal Experiences, Problems with Services, and Stigma/Effects on Family.

### Data analysis

Network analysis has received increasing attention in psychiatric research over the past years [33]. This approach includes graphical representations of the relationships between variables such as symptoms and provides the capacity to identify core features of complex networks. The network analysis was computed with RStudio [34] based on the methods described by Epskamp et al. 2018 [35]. Data and R-code are available online (https://osf.io/7vhxf/). In network models, variables are represented by ‘nodes’ connected by ‘edges’. All participants (*n* = 384) were included in the network analysis (there were no missing data for the CES-D, ZBI and BECI scales).

Two networks were estimated. The first network (Network 1a) consisted of the ZBI subscales (sum-scores): Negative Emotion/Consequences, Interpersonal Relationship, Time Demand, Patient’s Dependence, Self-accusation/guilt; the BECI subscales (sum-scores): Difficult Behaviours, Positive Personal Experiences, Problems with Services, Stigma/Effects on Family; and the total CES-D score (a total of 10 nodes). Data were normalized using the non-paranormal transformation [36]. The second network consisted of CES-D items in place of total CES-D score (Network 1b). The 20 CES-D items were first submitted to the *goldbricker* function from the R package *networktools* [37] comparing correlations in the network to identify nodes which most likely measure the same underlying construct (i.e., are co-linear). Two nodes (or variables) are deemed to be ‘redundant’ if the correlations between these two variables and all other variables are highly similar. If any, one variable is selected using the *net*_*reduce* function (see [37]). Two pairs were found redundant (see Results Sect. 3.2). Therefore, the second estimated network consisted of 27 nodes (i.e., ZBI: 5 subscales, BECI: 4 subscales, CES-D: 18 symptoms). Networks were estimated using the *estimateNetwork* function in the R package *bootnet* [38] with the “EBICglasso” method computing a Gaussian graphical model with the graphical LASSO [39] and extended Bayesian information criterion (EBIC; [40]) for model selection. In Gaussian graphical models, the parameters (i.e., edges) represent the association among two variables, after conditioning on all other variables in the network. The LASSO (‘least absolute shrinkage and selection operator’ [41]) is a regularization technique allowing parameters to be zero, resulting in a sparse network. The penalty parameter lambda (λ; the shrinkage parameter) was selected using the EBIC which involves the hyperparameter gamma (γ; we used the default value 0.5) to control the level of the penalization.

The centrality index *strength* was computed to quantify the role of nodes in the network (using *centrality* functions in the R package *qgraph* [42]). Strength indicates overall connections of each node and is calculated by summing the absolute edge weights that are connected to a specific node.

We estimated the accuracy of strength centrality indices using a case-dropping subset bootstrapping approach that determines how many cases can be removed from the network before the results become unstable, and estimated correlation stability coefficients (CS-coefficients). The CS represents the maximum proportion of population that can be dropped with re-calculated indices correlating at least 0.7 with indices of the initial sample. Networks with reliable centrality should have a CS ≥ 0.25, ideally ≥ 0.5. We also estimated the accuracy of edge-weights by calculating bootstrapped 95% confidence intervals (CIs) around the edge weights using 1,000 bootstraps.

## Results

### Sample characteristics

The characteristics of the sample are reported in Table 1. The mean age of caregivers was 61.8 years (standard deviation (SD) of 9.4 years). Most caregivers were women (73.7%), married (68.2%), and were caring for a child (87.5%). The mean age of relatives was 35.6 years (SD = 12.1), most of them being men (74%) and affected by schizophrenia or a schizoaffective disorder (71.9%). The mean total scores of the CES-D, ZBI, and BECI scales were, respectively, 18.2 (SD = 10.1), 38.1 (SD = 18.4), and 32.0 (SD = 12.3). Moderate-to-severe or severe burden (total ZBI score > 40) was reported in 44.3% subjects (*n* = 170), and 54.4% (*n* = 209) had a CES-D score ≥ 16, the cut-off value traditionally recommended for depression caseness [43], while 39.3% (*n* = 151) had a score of  ≥ 20, a value recently recommended for screening depression [44]. As expected, (Pearson’s) correlations between scales were high (CES-D–BECI: *r* = 0.51; CES-D–ZBI: *r* = 0.64; BECI–ZBI: *r* = 0.72). All descriptive statistics are reported in Supplementary Material Tables S1–S7.

**Table 1:** Characteristics of the sample (*n* = 384)

Variable	Descriptive statistics
Mean age (SD) [min–max]	61.8 (9.4) [24–84]
*Gender, n (%)*	
Women	283 (73.7)
Men	96 (25.0)
Missing values	5 (1.3)
*Marital status, n (%)*	
Married	262 (68.2)
Separated or divorced	73 (19.0)
Widowed	26 (6.8)
Single	18 (4.7)
Missing values	5 (1.3)
*Relationship to relative, n (%)*	
Parent	336 (87.5)
Partner	25 (6.5)
Sibling	14 (3.6)
Child	5 (1.3)
Other (unspecified)	4 (1.0)
*Relatives’ diagnosis, n (%)*	
Schizophrenia	227 (59.1)
Schizoaffective disorder	49 (12.8)
Bipolar disorder	46 (12.0)
Other (unspecified)	44 (11.5)
Unknown	45 (11.7)
Multiple	27 (7.0)
Mean age of relatives (SD) [min–max]	35.6 (12.1) [15–85]
*Gender of relative, n (%)*	
Women	99 (25.8)
Men	284 (74.0)
Missing value	1 (0.3)

### Network analysis

**Fig. 1:**
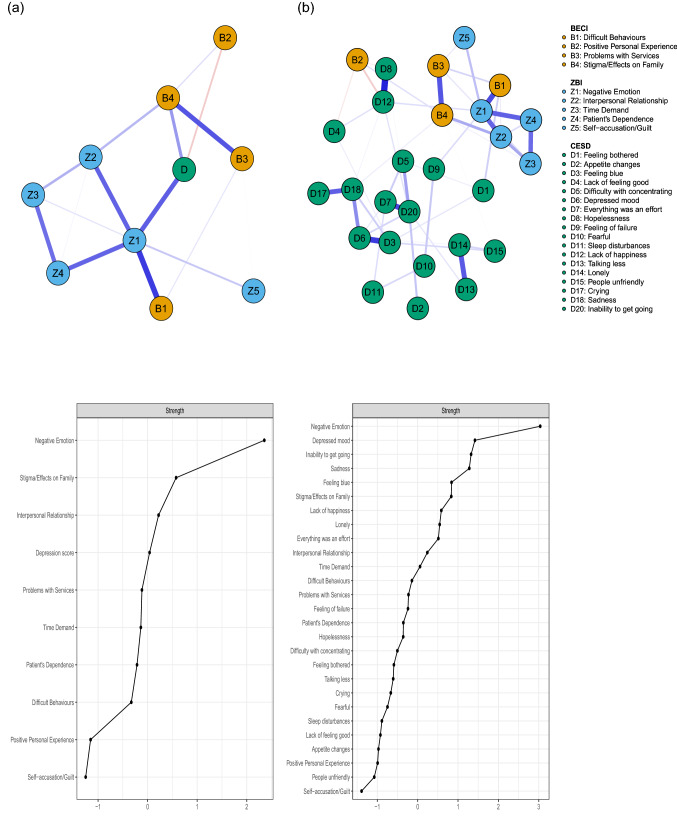
**a** Network displaying the relationships between total CES-D score, ZBI and BECI dimensions (i.e., subscales). **b** Network displaying the relationships between CES-D symptoms, ZBI and BECI dimensions. Blue and red edges represent positive and negative partial correlations between nodes, respectively. The thickness of the line indicates the strength of the association (i.e., the edge weight). Note that only estimates for which bootstrap 95% confidence interval did not contain zero are reported. Centrality (*Z*-scores) of each node, ranked by importance, is shown below the corresponding network

Estimated networks and nodes centrality are presented in Fig. 1. Before estimating Network 1b, two pairs of redundant nodes were identified: *lack of enjoyment* (item D16) with *lack of happiness* (item D12), and feeling *disliked by others* (item D19) with finding *people unfriendly* (item D15). *Lack of happiness*, and *people unfriendly* were kept in the network after the use of the *net*_*reduce* function.

The regularized partial correlations matrices are reported in Supplementary Material Tables S8 and S10. Bootstrap results are reported in Supplementary Material Tables S9 and S11 and shown in Fig. S1 and S3. Caregiving dimensions that connected the most to total depression score (Fig. 1a) were Negative Emotion/Consequences (Z1: *r* = 0.29), Stigma/Effects on Family (B4: *r* = 0.22), and Positive Personal Experience (B2: *r* = -0.16). In the second network (Fig. 1b), Negative Emotion/Consequences was predominantly associated with a *feeling of failure* (D9: *r* = 0.12), a *lack of happiness* (D12: *r* = 0.11), and a feeling that *everything was an effort* (D7: *r* = 0.07). Stigma/Effects on Family reliably connected to *sadness* (D18: *r* = 0.09) and finding *people unfriendly* (D15: *r* = 0.07), while (lack of) Positive Personal Experience was mainly associated with *lack of happiness* (D12: *r* = 0.13), *lack of feeling good* (D4: *r* = 0.10), and *hopelessness* (D8: *r* = 0.09). Of note, the BECI subscale Difficult Behaviours (B1) showed a stable association with *feeling bothered* (D1: *r* = 0.13).

The node with the highest standardized strength in Network 1a was Negative Emotion/Consequences (*Z* = 2.36), followed by Stigma/Effects on Family (*Z* = 0.58). Negative Emotion/Consequences remained the most central node in Network 1b (*Z* = 3.04). *Depressed mood* (D6: *Z* = 1.42), *inability to get going* (D20: *Z* = 1.32), *sadness* (D18: *Z* = 1.28), *feeling blue* (D4: *Z* = 0.84), and Stigma/Effects on Family (*Z* = 0.83) were among the most central nodes. Supplementary Material Fig. S2 and S4 show that strength centrality indices were stable (CS = 0.75 in Network 1a and 0.59 in Network 1b). In both networks, Negative Emotion/Consequences was, by far, the most central node.

### Untangling the negative emotion/consequences dimension

**Fig. 2:**
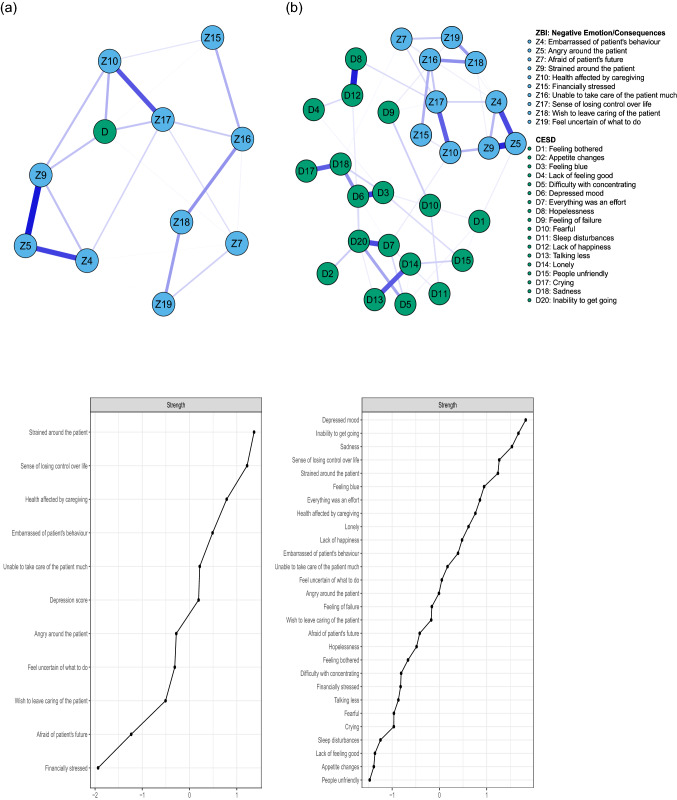
**a** Network displaying the relationships between total CES-D score and ZBI items from the Negative Emotion/Consequences dimension. **b** Network displaying the relationships between CES-D symptoms and ZBI items from the Negative Emotion/Consequences dimension. Edges represent positive partial correlations between nodes. The thickness of the line indicates the strength of the association. Note that only estimates for which 95% bootstrap confidence interval did not contain zero are reported. Centrality (*Z*-scores) of each node, ranked by importance, is shown below the corresponding network

A secondary set of networks were estimated to better understand the role of the Negative Emotion/Consequences component. Each item of this subscale was entered as a node, combined to the total CES-D score (Network 2a) or individual CES-D symptoms (Network 2b). Estimated networks and nodes centrality are presented in Fig. 2. The regularized partial correlations matrices are reported in Supplementary Material Tables S12 and S14 and bootstrap results are reported in Supplementary Material Tables S13 and S15 and shown in Figs. S5 and S7. Caregiving items that connected the most to total CES-D score (Fig. 2a) were *health affected by caregiving* (Z10: *r* = 0.19), feeling *strained around the patient* (Z9: *r* = 0.17), and a sense of *losing control over life* (Z17: *r* = 0.17). Being *afraid of patient’s future* (Z7: *r* = 0.11) and *financially stressed* (Z15: *r* = 0.11) also showed a stable association with depression severity. In regards with depressive symptoms (Fig. 2b), *health affected by caregiving* was predominantly associated with a feeling that *everything was an effort* (D7: *r* = 0.10). Feeling *strained around the patient* was mostly connected to *being fearful* (D10: *r* = 0.08), while a sense of *losing control over life* was reliably associated with both *hopelessness* (D8: *r* = 0.12) and a *feeling of failure* (D9: *r* = 0.09). Being *afraid of patient’s future* was mostly connected to *sadness* (D18: *r* = 0.09). Although *financial stress* showed edges with a few depressive symptoms (a *feeling of failure* (D9) resulting in the strongest estimate: *r* = 0.07), confidence intervals indicated a lower accuracy for these edges. However, feeling *angry around the patient* (Z5) was reliably associated with *feeling bothered* (D1: *r* = 0.10), feeling *unable to take care of the patient much* (Z16) with *lack of happiness* (D12: *r* = 0.08), and being *uncertain of what to do* (Z19) with a *feeling of failure* (D9: *r* = 0.10).

Similar to Network 1b, *depressed mood* (D6: *Z* = 1.83), *inability to get going* (D20: *Z* = 1.67), *sadness* (D18: *Z* = 1.54), and *feeling blue* (D4: Z = 0.95) were depressive symptoms with the highest standardized strength. A sense of *losing control over life* (Z17: *Z* = 1.27), feeling *strained around the patient* (Z9: *Z* = 1.24), and *health affected by caregiving* (Z10: *Z* = 0.76) were also among the most central nodes. Supplementary Material Fig. S6 and S8 show that strength centrality indices were stable (CS = 0.59 in both networks).

### Exploratory analysis: effect of psychoeducation

**Fig. 3:**
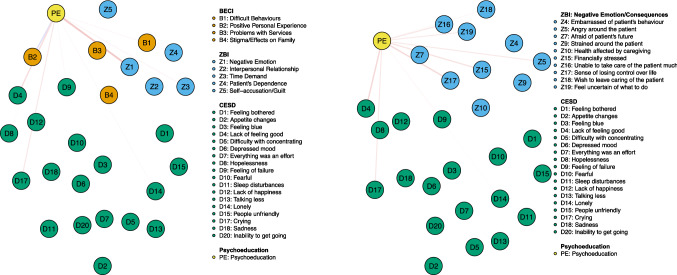
**a** Network displaying the relationships between psychoeducation, CES-D symptoms, ZBI and BECI dimensions (subscales). **b** Network displaying the relationships between psychoeducation, CES-D symptoms and ZBI items from the Negative Emotion/Consequences dimension. Blue and red edges represent positive and negative partial correlations between nodes, respectively. The thickness of the line indicates the strength of the relationship. Note that we only display and zoom in on relations between psychoeducation and other nodes for the sake of clarity

An (unplanned) independent-samples *t*-test indicated that CES-D total scores were significantly lower for subjects who followed a psychoeducational program (*n* = 221, M = 16.1, SD = 9.2) than for those who did not (*n* = 163, M = 21.0, SD = 10.6; *t*_(382)_ = 4.782, *p* < 0.001, Cohen’s *d* = 0.49, 95% CI 0.29–0.70). An exploratory set of networks including psychoeducation as a node (yes = 1 or no = 0) showed that small edges emerged between the latter and Negative Emotion/Consequences, (lack of) Positive Personal Experience, Time Demand, and Patient’s Dependence (*r*-values ranging from 0.01 to 0.08; see Fig. 3a). In the network including ZBI items from the Negative Emotion/Consequences dimension (Fig. 3b), psychoeducation was negatively associated with feeling *angry around the patient*, *financial stress*, *feeling unable to take care of the patient much*, *a sense of losing control over life*, *wishing to leave caring of the patient*, and being *uncertain of what to do* (*r*-values ranging from  – 0.01 to  – 0.06). Finally, in both networks, psychoeducation was negatively connected to *feeling blue*, *hopelessness*, a *feeling of failure*, *loneliness*, and *crying*. The regularized partial correlations matrices are reported in Supplementary Material Tables S16 and S17. Note that centrality and accuracy of edge-weights were not tested.

## Discussion

This study aimed to investigate associations between caregiving experiences and depression severity among caregivers of a relative living with a SMD. To the best of our knowledge, this is the first study to explore the multivariate structural dependencies among caregiving experiences and depressive symptomatology in a sample of family caregivers of individuals with a SMD, resulting in stable and meaningful relationships. Finally, an exploratory analysis identified emerging relationships between psychoeducation and depression symptoms or adverse caregiving experiences.

### Caregiving experiences associated with depression severity and its symptomatology

Caregiving-related dimensions or experiences conditionally associated with depression severity (i.e., after controlling for other variables) included Negative Emotion/Consequences (of caregiving), (lack of) Positive Personal Experience, and Stigma/Effects on Family (Fig. 1a). Among Negative Emotion/Consequences, *losing control over life*, *feeling strained around the relative* and *impaired self-perceived health* were of particular importance (Fig. 2a). Overall, these results are in line with both quantitative and qualitative studies that investigated family caregivers’ experiences of caring for relatives living with a SMD.

**Negative Emotion/Consequences** (of caregiving)—a ZBI subscale—was the most connected node to depression (CES-D score), while sharing an edge with all other ZBI subscales. In other words, the subscales Interpersonal Relationship, Time Demand, Patient's Dependence, and Self-accusation/Guilt were indirectly connected to depression through the Negative Emotion/Consequences component. The role of this dimension was further explored in subsequent analysis and discussed below. Depression score also shared a stable edge estimate with **(a lack of) Positive Personal Experience**—a BECI subscale comprising items such as *feeling confident in dealing with others* or *having discovered strengths in oneself*. Although the relationships between depression and Negative Emotion/Consequences and (a lack of) Positive Personal Experience in caregivers might be viewed as self-evident and axiomatic, it is interesting to note that in spite of sharing an edge with *lack of happiness*, they were nevertheless differentially associated with depressive symptoms. In fact, Negative Emotion/Consequences was predominantly associated with a *feeling of failure* and a feeling that *everything was an effort*, while (lack of) Positive Personal Experience essentially connected to *hopelessness*.

Perhaps a more insightful result relies on the conditional association between depression severity and **Stigma/Effects on Family** (BECI subscale), which appeared mostly associated with *sadness* and finding *people unfriendly*, the former being one of the most central nodes in estimated networks (and a core feature of depression). This set of results adds to and extends previous studies reporting that stigmatization, social isolation and disruption to family/household life are prevalent experiences among family caregivers of individuals with a SMD, and associated with stress and depression they might experience [45–47]. For instance, Chiou et al. (2009) [48] showed that perceived social support and perceived family function were negatively associated with burden. In a prospective study, Magliano et al. (2000) [49] reported a reduction of family burden at 1 year among relatives of patients with schizophrenia who received more practical support from their social network. Furthermore, perceived stigma has been shown to be positively associated with levels of depressive symptoms among caregivers of patients with a bipolar disorder [50]. Globally, these results suggest that interventions to caregivers should primarily target stigma and incorporate strategies on how to cope with the challenges posed to the family system and expand social support networks.

### Untangling the negative emotion/consequences dimension

***Feeling strained around the relative*, *a sense of losing control over life*** and ***impaired self-perceived health*** were the best correlates of depression severity in Network 2a (Fig. 2a) that aimed to better characterize the role of the Negative Emotion/Consequences subscale. With regard to their relationships with depressive symptoms (Fig. 2b), *feeling that one’s health is affected by caregiving* was predominantly associated with *feeling that everything was an effort* (a somatic component of depression). This is consistent with previous studies reporting that the amount of time spent for caregiving (per day) is related to caregiver’s burden or risk of depression [48, 51, 52]. It is thus not surprising that caregivers tend to self-perceive their health as poor or fairly poor compared to non-caregivers, schizophrenia caregivers being more likely to report sleep difficulties, insomnia, pain, and anxiety than other caregivers [53]. Our findings are also in accordance with the fact that poor physical health has been consistently identified as a significant correlate of depression in caregivers of persons with a SMD [22, 54–57], although this association is not limited to caregivers of individuals with a SMD [58]. Similarly, longitudinal studies, focusing on caregivers of a cognitively impaired adult, have suggested reciprocal relationships between depression and physical health [59, 60]. *Impaired self-perceived health* showed its strongest edge with ***a sense of losing control over life***, another item conditionally associated with depression severity and reliably connected to *hopelessness* and *a feeling of failure*. Altogether, these results suggest that the great deal of time and effort spent by caregivers in providing care to their ill relative (requiring numerous sacrifices with regard to their social and occupational life and physical needs) may significantly impair both their psychological wellbeing and physical health. They also corroborate the crucial relationship between psychological/physical health and depression in caregivers of subjects with a SMD. Although prospective studies in caregivers of individuals with a SMD are lacking, these results further highlight the need for service providers to pay special attention to the assessment of the health status of family caregivers.

**Feeling strained around the relative** also showed a stable association with depression severity. One can assume that family tension or stress experienced by caregivers might rely, at least in part, on relative’s symptoms and behaviors. Indeed, numerous studies carried out in caregivers of individuals with schizophrenia reported that clinical symptoms and behavioral issues were significant predictors of caregivers' depressive symptoms or burden [22, 55, 61–65]. In regards with specific relationships with CES-D symptoms, *feeling strained around the relative* was predominantly associated with being *fearful,* further supporting the hypothesis that relative’s behaviors may greatly contribute to the levels of tension that caregivers might experience when around their ill relative. In this regard, innovative strategies such as joint crisis plan could alleviate the negative impact of the relative’s symptoms and behaviors on caregivers [66].

Finally, being ***afraid of patient’s future*** and ***financially stressed*** were also connected to depression severity (Fig. 2a), showing their strongest edge weight with *sadness* and a *feeling of failure* respectively (Fig. 2b). Caregiving often includes financial support in terms of travel expenses, funding treatment and providing food; hence, many caregivers express difficulties in providing for their relative’s needs [47]. Our results are also consistent with studies reporting that lower income is associated with higher levels of depressive symptoms among caregivers of individuals with a SMD [22, 67, 68]. Such findings support the crucial importance of connecting caregivers to social workers so that they benefit from the financial assistance to which they are entitled.

### Network of depressive symptoms

In the estimated networks, the strongest associations involved CES-D symptoms: *lack of happiness—hopelessness*, *depressed mood—feeling blue*, *everything was an effort—inability to get going*, *talking less—loneliness*, *crying—sadness*, and *depressed mood—sadness*. All pairs but one (*talking less—loneliness*) involved items known to load on the same underlying first-order factor. For instance, the pairs *lack of happiness—hopelessness*, *depressed mood—feeling blue*, and *everything was an effort—inability to get going*, correspond, respectively, to the first-order factors ‘Positive Affect’, ‘Depressed Affect’, and ‘Somatic Complaint’ of the CES-D scale [28]. Our results are consistent with Santos et al. study (2007) [69] reporting the network structure of perinatal depressive symptoms in 515 Latina pregnant women. Indeed, the strongest edges identified in this latter study, namely, *lack of happiness*—*lack of enjoyment*, and *feeling like people were unfriendly*—*feeling disliked*, were found ‘redundant’ in our data (i.e., highly correlated and sharing a co-linear structure with the other variables). Moreover, similarly to our results, *crying*—*sadness*, and *sadness*—*depressed mood* were among the strongest reported associations [69]. These results suggest that part of symptom-to-symptom relationships that could drive caregiver depression processes might be shared with maternal depression. This raises the intriguing question whether some patterns of structural dependencies among depressive symptoms are relevant to various types of depression. Studies investigating the network structure of depressive symptoms in various populations are needed to address this issue.

Regarding symptom centrality, we found that *depressed mood*, *sadness*, *feeling blue*, *lack of happiness*, and *loneliness* were among the nodes with the highest strength. Correlation stability coefficient indicated good accuracy. Strikingly, all of these symptoms correspond to the first-order factor ‘Depressed Affect’ of the CES-D scale [28]. This is in line with the Santos et al. (2017) study [69] where the most central symptoms identified were *depressed mood*, *sadness*, *loneliness*, *feeling blue* and *lack of happiness*. However, this set of findings differ from the Santos et al. (2018) study [70] investigating the longitudinal network structure of depression symptoms in low-income depressed mothers where the strongest relationships were among *loneliness*—*sleep disturbance*, *inability to get going*—*crying*, and *concentration difficulty*—*feeling disliked*, these latter being the symptoms with highest strength centrality. One can hypothesize that differences across the studies might be explained, at least in part, by levels of depression severity which was higher in the Santos et al. 2018 study [70] compared to Santos et al. 2017 [69] and ours (M = 26.0, SD = 12.5, i.e., a 0.69 standard deviation difference with CES-D scores found here; a relatively large effect size). Remarkably, Santos et al. (2018) results [70] still showed similarities to ours: *inability to get going* and *feeling that everything was an effort* were also among the symptoms with the highest strength, further highlighting the importance of the somatic component in depression among caregivers.

### Psychoeducation

Depression scores were significantly lower for subjects who followed a psychoeducational program than for those who did not (Cohen’s *d* = 0.49, *i.e.,* a non-trivial difference). Although weak, a few associations emerged in exploratory networks incorporating psychoeducation as a node. First, consistently with previous studies reporting a significant positive effect of caregivers psychoeducation on depression over controls [71, 72], psychoeducation was negatively associated with Negative Emotion/Consequences, a *lack of feeling good*, *lack of happiness*, *crying*, *hopelessness* and *feeling of failure*. Second, psychoeducation was also negatively associated with Patient’s Dependence, Time Demand (3a), feeling *angry around the patient*, *unable to take care of the patient much*, and *uncertain of what to do* (3b). This is in accordance with the main goals of caregiver psychoeducation, i.e., providing illness education and problem-solving skills to enable caregivers to best assist their relative and cope with the severe challenges posed to the family system. Furthermore, psychoeducation was negatively associated with *feeling lonely* and with being *financially stressed*. This is consistent with the other major goal of caregivers psychoeducation which is to expand caregivers' social support networks by meeting peers and social workers [2]. In the same line, psychoeducation was associated with Positive Personal Experience that might rely on peer support interventions during which experiential coping strategies are shared. Importantly, a negative edge emerged between psychoeducation and a *sense of losing control over life*, one of the caregiving dimension that connected the most to depression severity and depressive symptoms.

While this set of results is broadly in line with literature reporting beneficial effects that psychoeducation may have on burden, depression, stress, and quality of life among caregivers of subjects with a SMD [73–76], they should be interpreted with caution as we did not collect any information about the type and duration of psychoeducational intervention received, as well as time since the intervention was provided. Based on these preliminary results, however, we believe that further studies are warranted to better characterize and understand how caregiver psychoeducation might target at and impact-specific depression symptoms and caregiving-related negative experiences. For instance, valuable studies would involve the investigation of *changes* in the network structure of burden dimensions associated with caregiving, and depression symptomatology after a psychoeducational program, as compared to initial measurement. Regarding caregiver psychoeducation, we believe that the present research could improve the content of new caregiver psychoeducational programs. Indeed, standardized psychoeducational programs with clear definitions of the essential content of interventions are currently lacking [77]. For instance, while it has been previously reported that caregiver psychoeducation is associated with a decrease in caregivers’ depressive symptomatology, which psychoeducational content is specifically associated with such a beneficial effect remains unknown. In this regard, we propose that the CES-D, ZARIT and BECI items or dimensions associated with caregiver psychoeducation in the presently identified network should be targeted primarily by the content of caregiver psychoeducation interventions. Such enhanced psychoeducational programs may help to reduce the risk of development or recurrence of caregivers' depression.

### Limitations

Some limitations should be mentioned. First, due to the cross-sectional design of our study, it is not possible to conclude for causal relationships between the identified predictors and the severity of depression. Longitudinal studies are needed to better explore this issue. Second, one should keep in mind that our data were collected during the first French global lockdown due to COVID-19. Previous reports have indicated increased depression rates in general population [82] and non-clinical samples [83–85] during lockdown. Here, 54.4% of caregivers presented a possible depressive disorder (CES-D total score ≥ 16), thus higher than the ones reported in caregivers of subjects with bipolar disorder or schizophrenia (respectively, 22–33% and 42%) outside lockdown periods [66, 79–81]. This figure is in line with Chiu et al. (2022) [78] reporting that 56% of family carers of older adults reported mild to severe depression between April and May 2020 (i.e., during lockdown in Hong Kong), a much higher prevalence than in the general population during the same period [79]. A few studies have shown exacerbated depression and burden during COVID-19, as compared to pre-pandemic levels, among caregivers of people with dementia [80] or with disability or cognitive decline [81]. Importantly, one study showed that caregivers had a greater likelihood of somatic and mental health issues than non-caregivers during the first months of the pandemic, even after adjusting for preexisting health status [82]. Undoubtedly, disruption of healthcare facilities and social restriction measures brought new challenges to caregivers facing an unexpected increase in responsibility and a greater experience of burden. In this context, psychological support interventions using digital solutions could be a useful format to improve the mental health of family caregivers [83]. Third, our results should be interpreted in the context of the particular scale used to evaluate depression and depressive features. Common depression scales differ substantially in symptom content [84]. Other depressive symptoms, not featured in the CES-D such as *somatic complaints*, might be relevant and should be investigated in future research carried out in caregivers. Fourth, our sample size did not allow for introducing socio-demographics such as gender, age, or marital status (among other characteristics reported in Table 1) into estimated networks. Although we used the bootstrapping methodology introduced by Epskamp et al. (2018) [35] for gaining insight into the accuracy of estimated parameters—resulting in meaningful and stable edges—, adding more nodes would have sizeably increased the number of estimated parameters, a threat to the accurate estimation of the models. However, caregiver’s factors including age, gender, educational level, income and patients’ factors such as age and clinical symptoms are likely to influence caregiving burden and depression levels on family caregivers [85]. For instance, higher income would decrease financial problem and stress related to providing care for ill family member [86]. Taking these covariates into account might impact the network structure of caregiving dimensions and depressive symptomatology estimated in the present study. Therefore, larger studies using a similar (network) approach are warranted to better characterize the inter-relationships between caregiving experiences, health-related outcomes including depression, *and* covariates. Finally, 83.9% of the caregivers included in the present study were members of family associations. Therefore, most of participants may have benefited from peer support and/or psychoeducational resources which are associated with lower depression and burden scores [73]. Studies assessing depression and burden in samples of caregivers who have never benefited from any caregiver interventions are needed to assess whether they are a more vulnerable subgroup. In this regard, surveys focusing on caregivers in early intervention services may be helpful.

## Conclusion

This study is the first to report the caregiving-related dimensions and experiences associated with depression severity among caregivers of a relative living with a SMD using a network approach. Characterization of the network structure of such caregiving experiences and depressive symptoms expands the knowledge regarding mental health of caregivers in psychiatry and allows a better understanding of the multivariate relationships underlying caregivers' depression symptomatology. In light of the complex nature of the identified network, we propose that caregivers' depression should be best addressed by the provision of various, complementary interventions including psychoeducation to caregivers. Expansion of this research topic can identify symptom-specific causal pathways and help to specify the topics that should be primarily addressed in caregiver psychoeducational interventions. Such enhanced psychoeducational programs may help to reduce the risk of development or recurrence of caregivers' depression.

## Supplementary Information

Below is the link to the electronic supplementary material.

Supplementary file1 (PDF 1171 KB)

## Data Availability

Data and R-code are available online (https://osf.io/7vhxf/).

## Code availability

Data and R-code are available online (https://osf.io/7vhxf/).
