# Supplementary material for: Caregiving-related experiences associated with depression severity and its symptomatology among caregivers of individuals with a severe mental disorder: an online cross-sectional study
Source: Eur Arch Psychiatry Clin Neurosci. 2022 Jun 30;273(4):887–900. doi: 10.1007/s00406-022-01451-3 (PMC9245882; doi:10.1007/s00406-022-01451-3)
Supplement: Supplementary file 1 — Supplementary file1 (PDF 1171 KB) [file 406_2022_1451_MOESM1_ESM.pdf]

Supplementary Material for the Article

Caregiving-related experiences associated with depression severity and its symptomatology among caregivers of individuals with a severe mental disorder: an online cross-sectional study

Louis-Ferdinand LESPINE[a,b] (PhD), Anne-Lise BOHEC[a,c] (PhD) Jean-Michel DOREY[d] (MD, PhD), Céline DUBIEN BERBEY[a] (PhD), Charles LOURIOUX[a], Thierry D'AMATO[a,c] (MD, PhD), Isabelle ROUCH[e] (MD, PhD), Marie-Odile KREBS (MD, PhD), Romain REY[a,c] (MD, PhD)

[a] University Lyon 1, Villeurbanne, France ; Schizophrenia Expert Center, Le Vinatier Hospital, Bron, France ; Fondation FondaMental, Créteil, France

[b] Service Universitaire d'Addictologie de Lyon, Le Vinatier Hospital, Bron, France

[c] INSERM, U1028; CNRS, UMR5292; Lyon Neuroscience Research Center, Psychiatric Disorders: from Resistance to Response Team, Lyon, France

[d] INSERM U1028, CNRS, UMR 5292, Lyon Neuroscience Research Center, EDUWELL, Lyon, France; Lyon 2 University ; Pôle PsyPA, Le Vinatier Hospital, Bron, France

[e] Neurology Unit, CM2R, CHU de Saint Etienne, France ; Clinical and Research Memory Center of Lyon, Villeurbanne, France; INSERM, U1219, Bordeaux Population Health Center, University of Bordeaux, France

CONTENTS

Table S1. Descriptive statistics of the Center for Epidemiologic Studies-Depression (CES-D) scale. N=384.
Table S2. Items of the Center for Epidemiologic Studies-Depression (CES-D).
Table S3. Descriptive statistics of the Zarit Burden Interview (ZBI). N=384.
Table S4. Zarit Burden Interview (ZBI).
Table S5. Descriptive statistics of the Brief Experience of Caregiving Inventory (BECI). N=384.
Table S6. Brief Experience of Caregiving Inventory (BECI).
Table S7. Pearson's correlations between scales (total scores). N=384.

Table S8. Partial correlations matrix of network 1a (Fig. 1a).
Fig S1. Bootstrap confidence intervals (CIs) of estimated edge-weights in network 1a.
Table S9. Summary of bootstrap results for network 1a.
Fig S2. Average correlations between centrality indices (strength) of networks sampled with persons dropped and the original sample (network 1a).

Table S10. Partial correlations matrix of network 1b (Fig. 1b).
Fig S3. Bootstrap confidence intervals (CIs) of estimated edge-weights in network 1b.
Table S11. Summary of bootstrap results for network 1b.
Fig S4. Average correlations between centrality indices (strength) of networks sampled with persons dropped and the original sample (network 1b).

Table S12. Partial correlations matrix of network 2a (Fig. 2a).
Fig S5. Bootstrap confidence intervals (CIs) of estimated edge-weights in network 2a.
Table S13. Summary of bootstrap results for network 2a.
Fig S6. Average correlations between centrality indices (strength) of networks sampled with persons dropped and the original sample (network 2a).

Table S14. Partial correlations matrix of network 2b (Fig. 2b).
Fig S7. Bootstrap confidence intervals (CIs) of estimated edge-weights in network 2b.
Table S15. Summary of bootstrap results for network 2b.
Fig. S8. Average correlations between centrality indices (strength) of networks sampled with persons dropped and the original sample (network 2b).

Table S16. Partial correlations matrix of network 3a (Fig. 3a).
Table S17. Partial correlations matrix of network 3b (Fig. 3b).

Table S1. Descriptive statistics of the Center for Epidemiologic Studies-Depression (CES-D) scale. N=384.

	Mean (SD) [min-max]	Median (IQR)
Total score	18.2 (10.1) [1-56]	17.0 (14.0)
	n (%)	
Total score ≥ 16	209 (54.4)	
Total score ≥ 20	151 (39.3)	

Table S2. Items of the Center for Epidemiologic Studies-Depression (CES-D).

Feeling bothered	D1
Appetite changes	D2
Feeling blue	D3
Lack of feeling good	D4
Difficulty with concentrating	D5
Depressed mood	D6
Everything was an effort	D7
Hopelessness	D8
Feeling of failure	D9
Fearful	D10
Sleep disturbances	D11
Lack of happiness	D12
Talking less	D13
Lonely	D14
People unfriendly	D15
Lack of enjoyment	D16
Crying	D17
Sadness	D18
Feeling disliked by others	D19
Inability to get going	D20

Table S3. Descriptive statistics of the Zarit Burden Interview (ZBI). N=384.

	Mean (SD) [min-max]	Median (IQR)
Total score (SD) [min-max]	38.1 (18.4) [0-85]	38.0 (25.25)
Negative Emotion<br>(items 4, 5, 7, 9, 10, 15, 16, 17, 18, 19)	17.4 (8.7) [0-40]	17.0 (12.25)
Interpersonal Relationship<br>(items 6, 11, 12, 13)	5.6 (4.5) [0-16]	5.0 (8.0)
Time Demand<br>(items 1, 2, 3)	5.0 (3.2) [0-12]	5.0 (4.0)
Patient’s Dependence<br>(items 8, 14)	5.2 (2.3) [0-8]	5.5 (3.0)
Self-accusation/Guilt<br>(items 20, 21)	2.9 (2.0) [0-8]	3.0 (2.0)
	n (%)	
Little or no burden (total score ≤ 20)	71 (18.5)	
Mild to moderate burden (total score: 21-40)	143 (37.2)	
Moderate to severe (total score: 41-60)	124 (32.3)	
Severe (total score >60)	46 (12.0)	

Table S4. Zarit Burden Interview (ZBI).

Patient asks for more help than he/she needs	ZBI 1
Not having enough time for yourself	ZBI 2
Stressed of fulfilling different responsibilities	ZBI 3
Embarrassed of patient behavior	ZBI 4
Feel angry around patient	ZBI 5
Negative effect on other relationships	ZBI 6
Afraid of patient’s future	ZBI 7
Patient is too dependent	ZBI 8
Feel strained around patient	ZBI 9
Health affected by caregiving	ZBI 10
Having inadequate privacy	ZBI 11
Suffering in social life	ZBI 12
Uncomfortable having friends	ZBI 13
Patient expected you to be the only caregiver	ZBI 14
Feel financially stressed	ZBI 15
Feel unable to take care of the patient much	ZBI 16
Sense of losing control over life	ZBI 17
Wish to leave caring of the patient	ZBI 18
Feel uncertain of what to do	ZBI 19
Feel should be doing more for the patient	ZBI 20
Feel could do better for the patient	ZBI 21
Feel burdened of caring	ZBI 22

Table S5. Descriptive statistics of the Brief Experience of Caregiving Inventory (BECI). N=384.

	Mean (SD) [min-max]	Median (IQR)
Total score (SD) [min-max]	32.0 (12.3) [1-60]	32.0 (18.0)
Difficult Behaviours (items 14, 15, 16, 17, 18, 19)	10.2 (5.7) [0-24]	10.0 (8.0)
Positive Personal Experience (items 4, 5, 7, 12)	8.4 (3.1) [0-15]	9.0 (5.0)
Problems with Services (items 2, 3, 6, 8, 11)	7.6 (4.8) [0-20]	7.0 (7.0)
Stigma/Effects on Family (items 1, 9, 10, 13)	5.7 (3.6) [0-16]	5.0 (5.0)

Table S6. Brief Experience of Caregiving Inventory (BECI).

Feeling unable to tell anyone about his illness	BECI 1
How mental health professionals do not take you seriously	BECI 2
Dealing with psychiatrists	BECI 3
I have become more confident dealing with others	BECI 4
I have become more understanding of others with problems	BECI 5
How to deal with mental health professionals	BECI 6
He/she is good company	BECI 7
How health professionals do not understand your situation	BECI 8
How to explain his illness to others	BECI 9
Others leaving home because of the effects of the illness	BECI 10
How to make complaints about his care	BECI 11
I have discovered strengths in myself	BECI 12
The illness causing a family breakup	BECI 13
(Patient being) Unpredictable	BECI 14
(Patient being) Uncommunicative	BECI 15
(Patient being) Not interested	BECI 16
(Patient being) Irritable	BECI 17
(Patient being) Inconsiderate	BECI 18
Patient behaving in a reckless way	BECI 19

Table S7. Pearson's correlations between scales (total scores). N=384. *p*-values < 0.001.

	CESD	ZBI	BECI
CESD	—	—	—
ZBI	.638	—	—
BECI	.512	.717	—

Table S8. Partial correlations matrix of network 1a (Fig. 1a). Boxes highlighted in blue indicate edges for which 95% bootstrap confidence intervals did not contain zero. Dark blue indicate edges between BECI/ZBI dimensions and total CES-D score (D).

	B1	B2	B3	B4	Z1	Z2	Z3	Z4	Z5	D
B1										
B2	0									
B3	0.13	0.08								
B4	0.02	0.14	0.30							
Z1	0.33	-0.02	0.10	0.04						
Z2	0.09	0	0	0.19	0.28					
Z3	0.07	0	0	0.10	0.13	0.19				
Z4	0.05	0	0.02	0	0.28	0.11	0.27			
Z5	0	0	0.10	0.02	0.17	0	0	0.004		
D	0.01	-0.16	0.04	0.22	0.29	0.03	0	0	0.08	

B1=Difficult Behaviours; B2=Positive Personal Experience; B3=Problems with Services; B4=Stigma/Effects on Family. Z1=Negative Emotion/Consequences; Z2=Interpersonal Relationships; Z3=Time Demand; Z4=Patient's Dependence; Self-accusation/Guilt. D=Depression (total CESD score).

Fig S1. Bootstrap confidence intervals (CIs) of estimated edge-weights in network 1a. The red line indicates the sample values and the grey area the bootstrap CIs. Each horizontal line represents one edge of the network, ordered from the edge with the highest weight to the edge with the lowest weight. In the case of ties (for instance, multiple edge-weights were estimated to be exactly 0), the mean of the *bootstrap* samples was used in ordering the edges. Boxes indicate the proportion of times parameters were estimated to be zero.

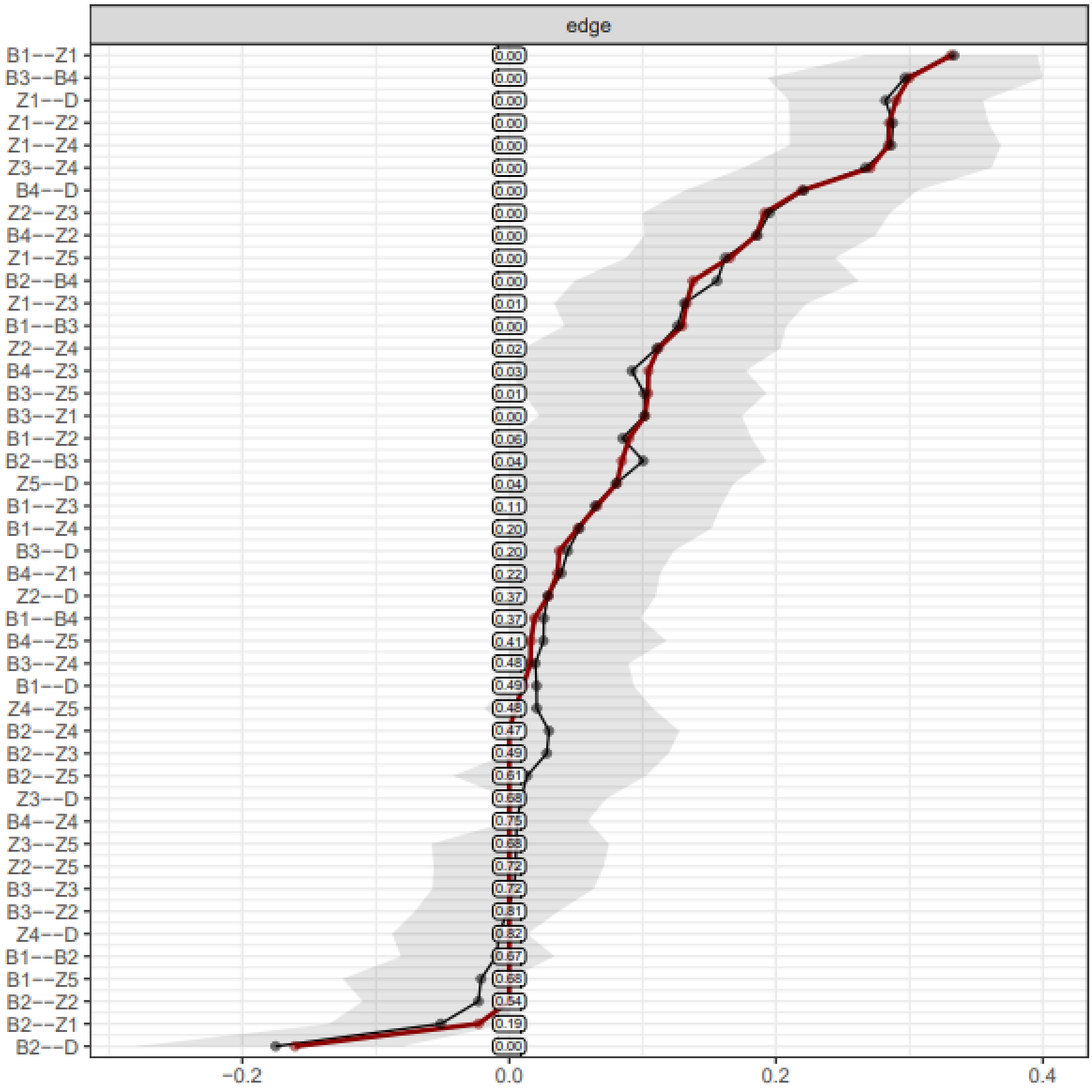

Table S9. Summary of bootstrap results for network 1a (only edges for which 95% bootstrap confidence intervals did not contain zero are reported: 18 edges over 45 estimated edges). Edges are ranked by absolute edge weight. Dark blue indicate edges between BECI/ZBI dimensions and total CES-D score (D). "prop0" indicates the proportion of times parameters were estimated to be zero.

		Bootstrap results			
Edge	Sample	Mean	CI lower	CI upper	prop0
B1--Z1	0.33	0.33	0.27	0.40	0
B3--B4	0.30	0.30	0.20	0.40	0
Z1--D	0.29	0.28	0.22	0.36	0
Z1--Z2	0.28	0.29	0.21	0.36	0
Z1--Z4	0.28	0.29	0.20	0.36	0
Z3--Z4	0.27	0.27	0.17	0.37	0
B4--D	0.22	0.22	0.13	0.31	0
Z2--Z3	0.19	0.20	0.10	0.28	0
B4--Z2	0.19	0.19	0.10	0.27	0
Z1--Z5	0.17	0.16	0.08	0.25	0
B2--D	-0.16	-0.18	-0.26	-0.06	0
B2--B4	0.14	0.16	0.03	0.24	0.4
Z1--Z3	0.13	0.13	0.04	0.23	0.6
B1--B3	0.13	0.13	0.04	0.22	0.3
Z2--Z4	0.11	0.11	0.02	0.21	1.6
B4--Z3	0.10	0.09	0.02	0.19	3.3
B3--Z5	0.10	0.10	0.01	0.20	1.3
B3--Z1	0.10	0.10	0.02	0.18	0.3

B1=Difficult Behaviours; B2=Positive Personal Experience; B3=Problems with Services; B4=Stigma/Effects on Family. Z1=Negative Emotion/Consequences; Z2=Interpersonal Relationships; Z3=Time Demand; Z4=Patient's Dependence; Self-accusation/Guilt. D=Depression (total CESD score).

Fig. S2. Average correlations between centrality indices (strength) of networks sampled with persons dropped and the original sample (network 1a). The line indicates the means and areas indicate the range from the 2.5th to the 97.5th quantiles. Maximum drop proportions to retain correlation of 0.7 in at least 95% of the samples: 0.75 (maximum proportion tested).

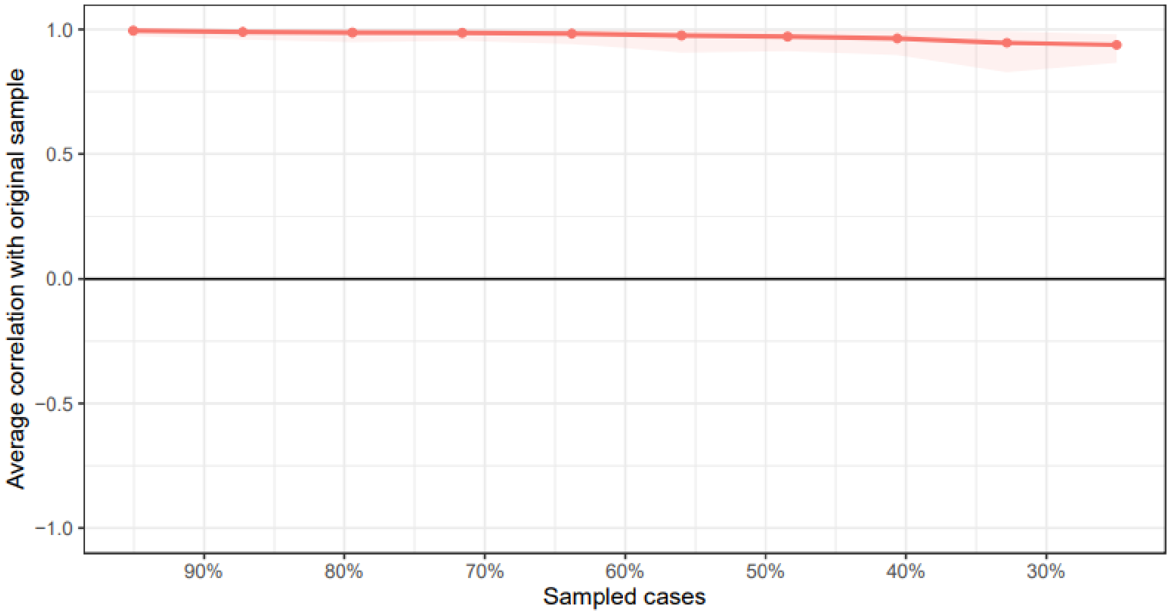

Table S10. Partial correlations matrix of network 1b (Fig. 1b). Boxes highlighted in blue indicate edges for which 95% bootstrap confidence intervals did not contain zero. Dark blue indicate edges between BECI/ZBI dimensions and CES-D symptoms.

	B1	B2	B3	B4	Z1	Z2	Z3	Z4	Z5	D1	D2	D3	D4	D5	D6	D7	D8	D9	D10	D11	D12	D13	D14	D15	D17	D18	D20
B1																											
B2	0																										
B3	0.12	0.07																									
B4	0.02	0.11	0.29																								
Z1	0.30	0	0.10	0.06																							
Z2	0.09	0	0	0.18	0.27																						
Z3	0.05	0	0	0.09	0.12	0.18																					
Z4	0.06	0	0.02	0	0.28	0.12	0.26																				
Z5	0	0	0.10	0.01	0.15	0	0	0.004																			
D1	0.13	0	0	0	0.05	0	0.08	0	0																		
D2	0.03	0	0	0	0	0	0	0	0	0.01																	
D3	0	0	0	0	0	0	0	0	0	0.10	0.09																
D4	0	-0.10	0	0	0	0	0	-0.01	0	0	0	0.09															
D5	0	0	0.001	0.05	0.02	0	0	0	0.03	0.09	0.03	0	0														
D6	0	0	0	0	0.04	0	0	0	0	0.04	0.01	0.32	0.04	0.03													
D7	0	0	0	0.005	0.07	0.003	0	0	0	0.04	0.04	0.01	0	0.13	0.10												
D8	0	-0.09	0	0.01	0.03	0.02	0	0	0	0	0	0.04	0.04	0.001	0.02	0											
D9	0	-0.04	0	0.003	0.12	0	0	0	0.06	0	0	0.07	0.05	0	0.04	0.01	0.05										
D10	0	0	0	0	0.04	0	0	0	0	0	0.01	0	0	0.02	0.08	0.01	0.01	0.14									
D11	0	0	0	0.04	0	0	0.03	0	0	0	0.06	0	0	0.03	0.05	0.11	0.01	0	0.12								
D12	0	-0.13	0.01	0	0.11	0.02	0	0	0.01	0.01	0.01	0	0.11	0	0	0	0.36	0.05	0	0							
D13	0	0	0	0	0	0	0	0	0	0.02	0.08	0.01	0.04	0	0	0.01	0	0.02	0.05	0.03	0.01						
D14	0	0	0.03	0.07	0.03	0.04	0.03	0	0.02	0	0.02	0.03	0.004	0.08	0.08	0.06	0	0.02	0	0.00	0.01	0.28					
D15	0	0	0.002	0.07	0	0	0.02	0	0	0.08	0	0.12	0	0	0	0.03	0	0	0.07	0	0	0.005	0.12				
D17	0	0	0.04	0	0	0	0	0	0	0	0.03	0.08	0.07	0	0.09	0	0	0.05	0	0	0	0	0.02	0			
D18	0	0	0	0.09	0.003	0	0	0	0.04	0	0	0.13	0	0	0.22	0.06	0	0.07	0.08	0.05	0.08	0	0.06	0	0.28		
D20	0	-0.004	0	0	0	0	0	0	0.01	0.02	0.14	0.004	0.01	0.19	0.13	0.31	0.08	0.01	0.01	0.04	0.11	0.10	0	0	0	0.08	

B1=Difficult Behaviours; B2=Positive Personal Experience; B3=Problems with Services; B4=Stigma/Effects on Family. Z1=Negative Emotion/Consequences; Z2=Interpersonal Relationships; Z3=Time Demand; Z4=Patient's Dependence; Self-accusation/Guilt. D1=feeling bothered; D2=appetite changes; D3=feeling blue; D4=lack of feeling good; D5=difficulty with concentrating; D6=depressed mood; D7=everything was an effort; D8=hopelessness; D9=feeling of failure; D10=fearful; D11=sleep disturbances; D12=lack of happiness; D13=talking less; D14= lonely; D15=people unfriendly; D17=crying. D18=sadness; D20=inability to get going.

Fig S3. Bootstrap confidence intervals (CIs) of estimated edge-weights in network 1b. The red line indicates the sample values and the grey area the bootstrap CIs. Each horizontal line represents one edge of the network, ordered from the edge with the highest weight to the edge with the lowest weight. In the case of ties (for instance, multiple edge-weights were estimated to be exactly 0), the mean of the *bootstrap* samples was used in ordering the edges. The y-axis labels have been removed to avoid cluttering.

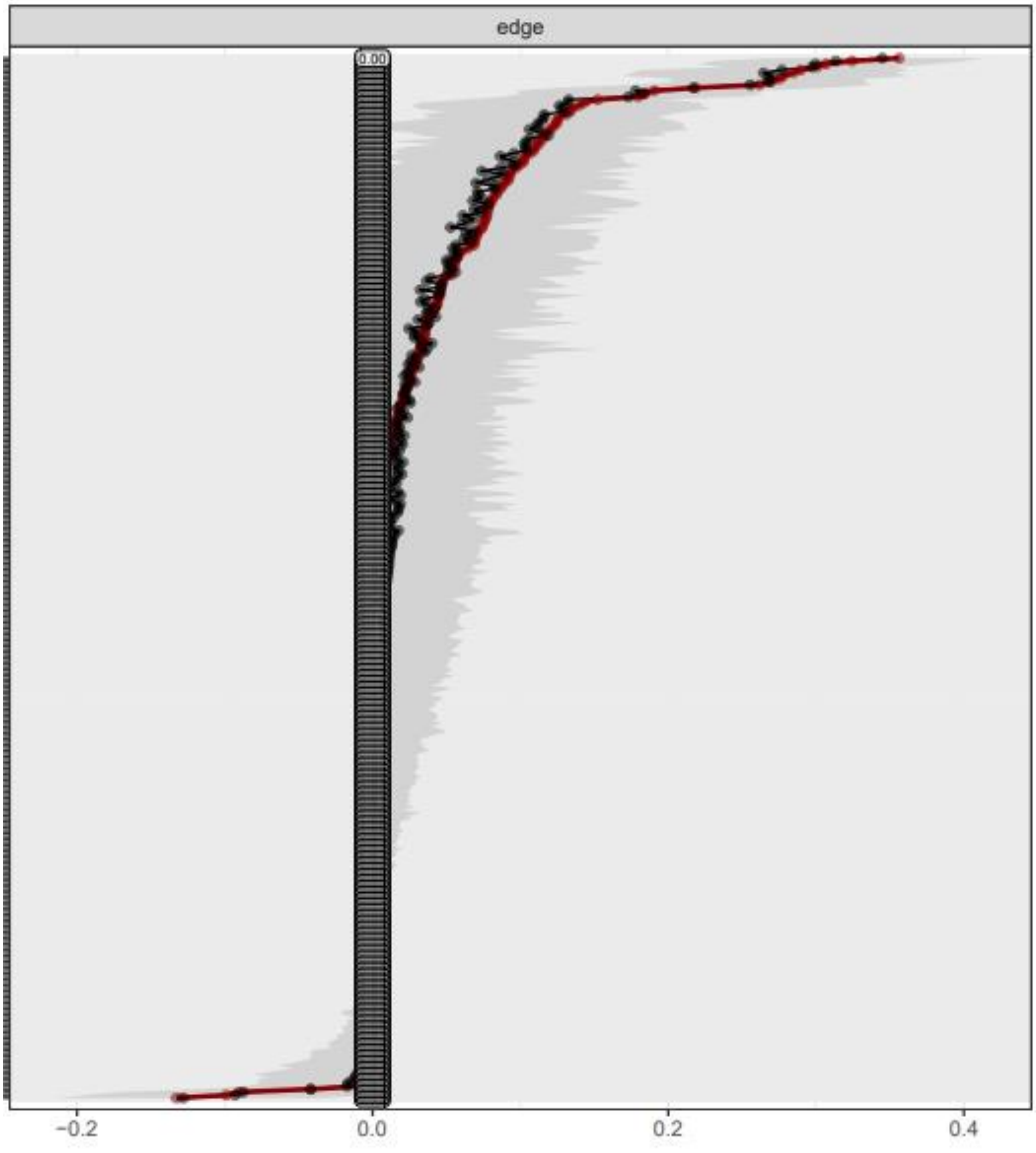

Table S11. Summary of bootstrap results for network 1b (only edges for which 95% bootstrap confidence intervals did not contain zero are reported: 50 edges over 352 estimated edges). Edges are ranked by absolute edge weight. Dark blue indicate edges between BECI/ZBI dimensions and CES-D items. "prop0" indicates the proportion of times parameters were estimated to be zero.

		Bootstrap results			
Edge	Sample	Mean	CI lower	CI upper	prop0
D8--D12	0.36	0.34	0.28	0.43	0
D3--D6	0.32	0.31	0.24	0.41	0
D7--D20	0.31	0.30	0.23	0.39	0
B1--Z1	0.30	0.30	0.24	0.36	0
B3--B4	0.29	0.28	0.20	0.38	0
D13--D14	0.28	0.26	0.20	0.37	0
D17--D18	0.28	0.27	0.18	0.38	0
Z1--Z4	0.28	0.27	0.21	0.34	0
Z1--Z2	0.27	0.27	0.20	0.34	0
Z3--Z4	0.26	0.26	0.18	0.35	0
D6--D18	0.22	0.22	0.13	0.31	0
D5--D20	0.19	0.18	0.11	0.27	0
Z2--Z3	0.18	0.18	0.10	0.27	0
B4--Z2	0.18	0.17	0.10	0.26	0
Z1--Z5	0.15	0.13	0.08	0.22	0
D2--D20	0.14	0.13	0.05	0.24	0.2
D9--D10	0.14	0.13	0.04	0.24	0.5
D5--D7	0.13	0.13	0.03	0.24	0.8
D3--D18	0.13	0.13	0.05	0.22	0.2
B2--D12	-0.13	-0.13	-0.22	-0.04	0.3
B1--D1	0.13	0.12	0.05	0.22	0.4
D6--D20	0.13	0.12	0.05	0.20	0.2
B1--B3	0.12	0.11	0.04	0.21	0.6
D14--D15	0.12	0.11	0.02	0.23	2.2
D10--D11	0.12	0.11	0.02	0.23	1.7
D3--D15	0.12	0.11	0.01	0.23	3.1
Z2--Z4	0.12	0.11	0.03	0.20	0.5
Z1--Z3	0.12	0.12	0.03	0.20	0.2
Z1--D9	0.12	0.11	0.05	0.18	0.1
D7--D11	0.11	0.10	0.02	0.20	1.4
Z1--D12	0.11	0.10	0.05	0.18	0.1
B2--B4	0.11	0.10	0.03	0.19	0.9
D4--D12	0.11	0.10	0.02	0.20	1.8
D12--D20	0.11	0.10	0.03	0.18	0.9
D13--D20	0.10	0.09	0.02	0.18	1.7
D1--D3	0.10	0.09	0.02	0.18	1.6
B3--Z1	0.10	0.10	0.03	0.18	0.4
D6--D7	0.10	0.10	0.01	0.18	1.3
B2--D4	-0.10	-0.09	-0.20	-0.002	4.2
B3--Z5	0.10	0.09	0.01	0.18	2.6
B4--D18	0.09	0.07	0.02	0.17	3.1
B1--Z2	0.09	0.09	0.005	0.18	2.2
D3--D4	0.09	0.08	0.02	0.16	1.4
B2--D8	-0.09	-0.09	-0.18	-0.003	3
B4--Z3	0.09	0.08	0.01	0.17	2.4
D12--D18	0.08	0.07	0.01	0.15	3.1
D6--D14	0.08	0.07	0.01	0.16	4.2
D8--D20	0.08	0.06	0.01	0.15	5.9
B4--D15	0.07	0.06	0.003	0.15	6.6
Z1--D7	0.07	0.05	0.01	0.13	5

B1=Difficult Behaviours; B2=Positive Personal Experience; B3=Problems with Services; B4=Stigma/Effects on Family. Z1=Negative Emotion/Consequences; Z2=Interpersonal Relationships; Z3=Time Demand; Z4=Patient's Dependence; Self-accusation/Guilt. D1=feeling bothered; D2=appetite changes; D3=feeling blue; D4=lack of feeling good; D5=difficulty with concentrating; D6=depressed mood; D7=everything was an effort; D8=hopelessness; D9=feeling of failure; D10=fearful; D11=sleep disturbances; D12=lack of happiness; D13=talking less; D14= lonely; D15=people unfriendly; D17=crying. D18=sadness; D20=inability to get going.

Fig. S4. Average correlations between centrality indices (strength) of networks sampled with persons dropped and the original sample (network 1b). The line indicates the means and areas indicate the range from the 2.5th to the 97.5th quantiles. Maximum drop proportions to retain correlation of 0.7 in at least 95% of the samples: 0.594.

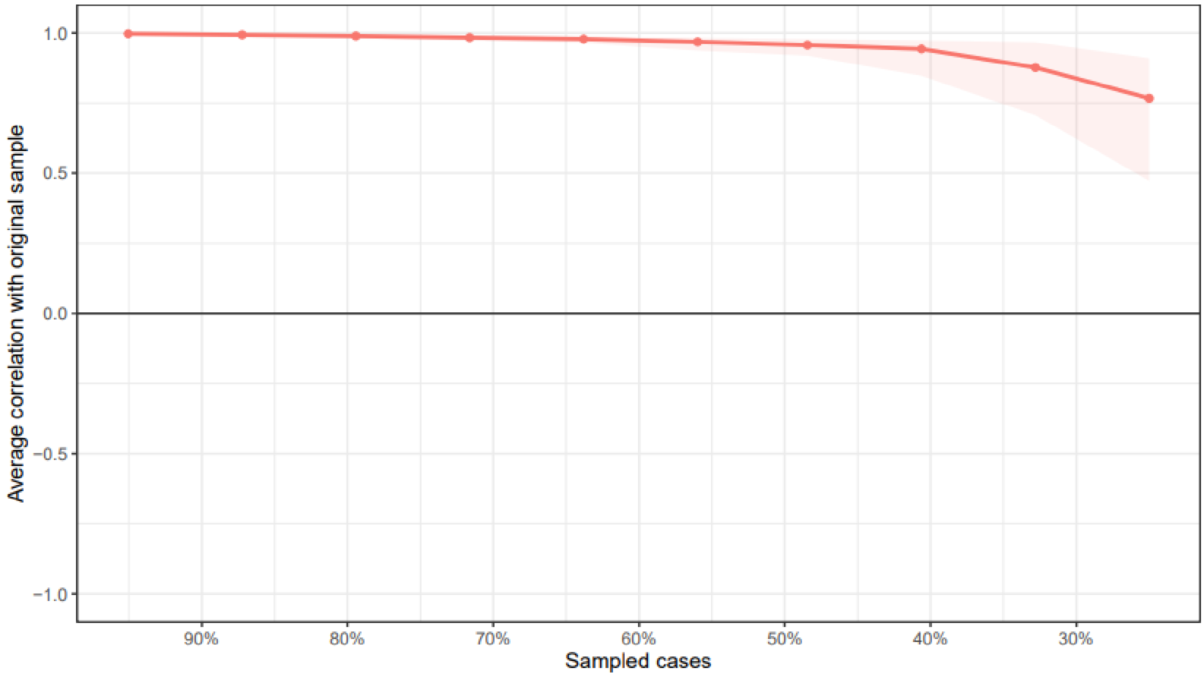

Table S12. Partial correlations matrix of network 2a (Fig. 2a). Boxes highlighted in blue indicate edges for which 95% bootstrap confidence interval did not contain zero. Dark blue indicate edges between ZBI items (Negative Emotion/Consequences dimension) and total CES-D score (D).

	Z4	Z5	Z7	Z9	Z10	Z15	Z16	Z17	Z18	Z19	D
Z4											
Z5	0.32										
Z7	0.11	0									
Z9	0.18	0.37	0								
Z10	0	0	0	0.18							
Z15	0	0	0.06	0	0.13						
Z16	0	0.02	0.11	0	0.06	0.19					
Z17	0.15	0.02	0.14	0.01	0.30	0.06	0.16				
Z18	0.09	0.03	0	0.10	0.07	0.02	0.23	0.04			
Z19	0.10	0.06	0.15	0.07	0.07	0	0.06	0.00	0.22		
D	0	0	0.11	0.17	0.19	0.11	0.06	0.17	0	0.09	

Z4=Embarrassed of patient's behaviour; Z5=Angry around the patient Z7=Afraid of patient's future; Z9=Strained around the patient; Z10=Health affected by caregiving; Z15=Financially stressed; Z16=Unable to take care of the patient much; Z17=Sense of losing control over life; Z18=Wish to leave caring of the patient; Z19=Feel uncertain of what to do. D=Depression (total CESD score).

Fig S5. Bootstrap confidence intervals (CIs) of estimated edge-weights in network 2a. The red line indicates the sample values and the grey area the bootstrap CIs. Each horizontal line represents one edge of the network, ordered from the edge with the highest weight to the edge with the lowest weight. In the case of ties (for instance, multiple edge-weights were estimated to be exactly 0), the mean of the *bootstrap* samples was used in ordering the edges. Boxes indicate the proportion of times parameters were estimated to be zero.

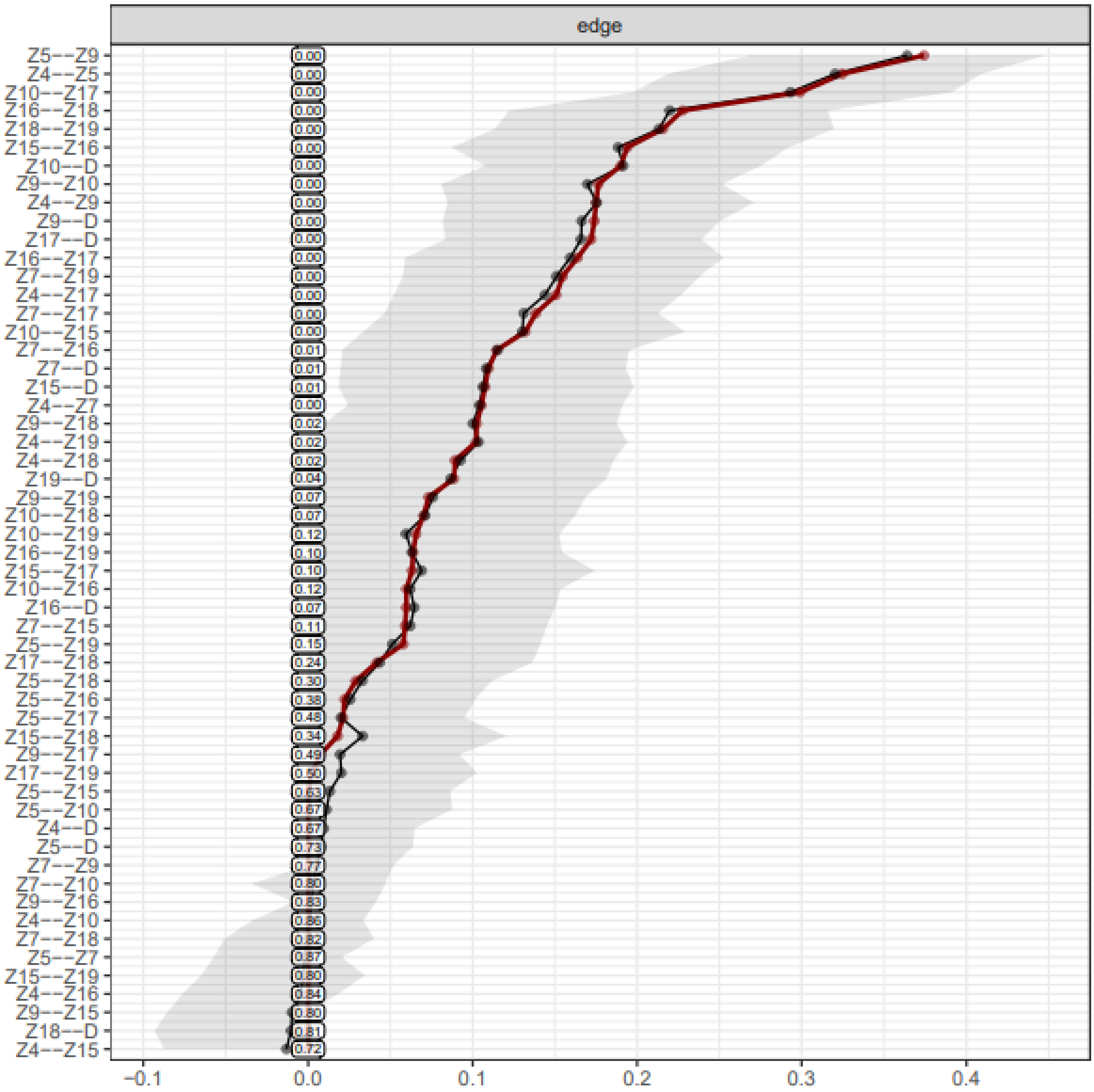

Table S13. Summary of bootstrap results for network 2a (only edges for which 95% bootstrap confidence interval did not contain zero are reported: 22 edges over 55 estimated edges). Edges are ranked by absolute edge weight. Dark blue indicate edges between ZBI and CES-D items. "prop0" indicates the proportion of times parameters were estimated to be zero.

		Bootstrap results			
Edge	Sample	Mean	CI lower	CI upper	prop0
Z5--Z9	0.37	0.36	0.28	0.46	0
Z4--Z5	0.32	0.32	0.23	0.42	0
Z10--Z17	0.30	0.29	0.20	0.40	0
Z16--Z18	0.23	0.22	0.13	0.33	0
Z18--Z19	0.22	0.21	0.11	0.32	0
Z10--D	0.19	0.19	0.10	0.28	0
Z17--D	0.17	0.17	0.09	0.25	0
Z9--Z10	0.18	0.17	0.09	0.26	0
Z15--Z16	0.19	0.19	0.09	0.30	0.1
Z9--D	0.17	0.17	0.09	0.26	0
Z4--Z9	0.18	0.18	0.08	0.27	0
Z7--Z19	0.15	0.15	0.06	0.25	0
Z16--Z17	0.16	0.16	0.06	0.26	0.1
Z4--Z17	0.15	0.14	0.06	0.24	0
Z7--Z17	0.14	0.13	0.06	0.22	0.1
Z10--Z15	0.13	0.13	0.03	0.23	0.2
Z7--Z16	0.11	0.12	0.02	0.21	0.9
Z7--D	0.11	0.11	0.02	0.20	0.6
Z4--Z7	0.11	0.10	0.02	0.19	0.3
Z15--D	0.11	0.11	0.02	0.20	1.3
Z9--Z18	0.10	0.10	0.01	0.19	1.6
Z4--Z19	0.10	0.10	0.01	0.19	1.7

Z4=Embarrassed of patient's behaviour; Z5=Angry around the patient Z7=Afraid of patient's future; Z9=Strained around the patient; Z10=Health affected by caregiving; Z15=Financially stressed; Z16=Unable to take care of the patient much; Z17=Sense of losing control over life; Z18=Wish to leave caring of the patient; Z19=Feel uncertain of what to do. D=Depression (total CESD score).

Fig. S6. Average correlations between centrality indices (strength) of networks sampled with persons dropped and the original sample (network 2a). The line indicates the means and areas indicate the range from the 2.5th to the 97.5th quantiles. Maximum drop proportions to retain correlation of 0.7 in at least 95% of the samples: 0.594.

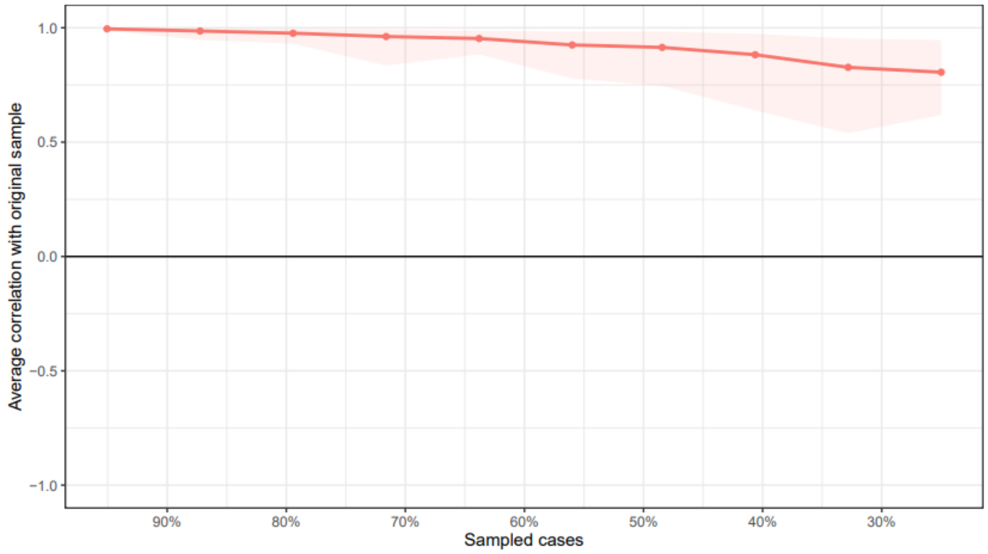

Table S14. Partial correlations matrix of network 2b (Fig. 2b). Boxes highlighted in blue indicate edges for which 95% bootstrap confidence intervals did not contain zero. Dark blue indicate edges between ZBI items (Negative Emotion/Consequences dimension) and CESD items.

	Z4	Z5	Z7	Z9	Z10	Z15	Z16	Z17	Z18	Z19	D1	D2	D3	D4	D5	D6	D7	D8	D9	D10	D11	D12	D13	D14	D15	D17	D18	D20
Z4																												
Z5	0.31																											
Z7	0.10	0																										
Z9	0.17	0.35	0																									
Z10	0	0	0	0.17																								
Z15	0	0	0.05	0	0.11																							
Z16	0	0.01	0.11	0	0.05	0.18																						
Z17	0.14	0.01	0.12	0.02	0.28	0.05	0.15																					
Z18	0.09	0.02	0	0.10	0.07	0.01	0.22	0.04																				
Z19	0.09	0.05	0.15	0.08	0.06	0	0.06	0	0.21																			
D1	0.03	0.10	0	0.03	0.001	0	0	0.03	0.06	0.02																		
D2	0	0	0	0	0.01	0	0	0	0	0	0.02																	
D3	0	0	0	0	0	0	0	0	0	0	0.10	0.09																
D4	0	0	-0.05	0	0	0	0	0	0	0	0	0	0.09															
D5	0	0	0	0.03	0.05	0.01	2E-04	0	0	0	0.10	0.03	0	0														
D6	0	0	0.02	0	0.07	0	0	0.03	0	0	0.04	0.004	0.33	0.04	0.02													
D7	0	0	0	0.02	0.10	0.04	0.02	0.01	0	0	0.04	0.04	0.01	0	0.13	0.09												
D8	0	0	0.08	0	0	0	0	0.12	0	0.01	0	0	0.03	0.05	0	0	0											
D9	0.02	0.01	0	0	0	0.07	0.002	0.09	0	0.10	0	0	0.07	0.06	0	0.03	0.004	0.03										
D10	0	0	0	0.08	0	0.002	0	0	0	0	0	0.01	0	0	0.02	0.07	0.01	0.003	0.14									
D11	0	0	0.01	0.03	0.01	0.04	0	0	0	0	0	0.06	0	0	0.03	0.05	0.11	0.005	0	0.12								
D12	0	0.01	0	0.07	0.02	0	0.08	0.02	0	0.03	0.01	0.02	0	0.13	0	0	0	0.36	0.06	0	0							
D13	0.01	0	0	0	0	0	0	0	0	0	0.03	0.08	0.01	0.05	0	0	0.01	0	0.01	0.05	0.03	0.01						
D14	0	0	0	0	0.05	0.06	0	0.07	0	0.03	0	0.02	0.03	0.003	0.09	0.08	0.06	0	0.01	0	0.01	0.004	0.29					
D15	0.01	0	0	0	0	0.06	0	0	0	0	0.09	0	0.13	0	0	0	0.03	0	0	0.07	0	0	0.01	0.14				
D17	0	0	0	0	0.01	0	0	0	0	0.02	0	0.03	0.08	0.07	0	0.08	0	-0.01	0.04	0	0	0	0	0.02	0			
D18	0	0	0.09	0.02	0	0	0.01	0	0	0	0	0	0.13	0	0	0.22	0.06	0	0.07	0.07	0.05	0.08	0	0.07	0	0.28		
D20	0	0	0	0	0	0	0.02	0	-0.02	0	0.02	0.14	0.003	0.01	0.19	0.13	0.31	0.07	0.01	0.01	0.04	0.11	0.10	0	0	0	0.08	

Z4=Embarrassed of patient's behaviour; Z5=Angry around the patient Z7=Afraid of patient's future; Z9=Strained around the patient; Z10=Health affected by caregiving; Z15=Financially stressed; Z16=Unable to take care of the patient much; Z17=Sense of losing control over life; Z18=Wish to leave caring of the patient; Z19=Feel uncertain of what to do. D1=feeling bothered; D2=appetite changes; D3=feeling blue; D4=lack of feeling good; D5=difficulty with concentrating; D6=depressed mood; D7=everything was an effort; D8=hopelessness; D9=feeling of failure; D10=fearful; D11=sleep disturbances; D12=lack of happiness; D13=talking less; D14= lonely; D15=people unfriendly; D17=crying. D18=sadness; D20=inability to get going.

Fig S7. Bootstrap confidence intervals (CIs) of estimated edge-weights in network 2b. The red line indicates the sample values and the grey area the bootstrap CIs. Each horizontal line represents one edge of the network, ordered from the edge with the highest weight to the edge with the lowest weight. In the case of ties (for instance, multiple edge-weights were estimated to be exactly 0), the mean of the *bootstrap* samples was used in ordering the edges. The y-axis labels have been removed to avoid cluttering.

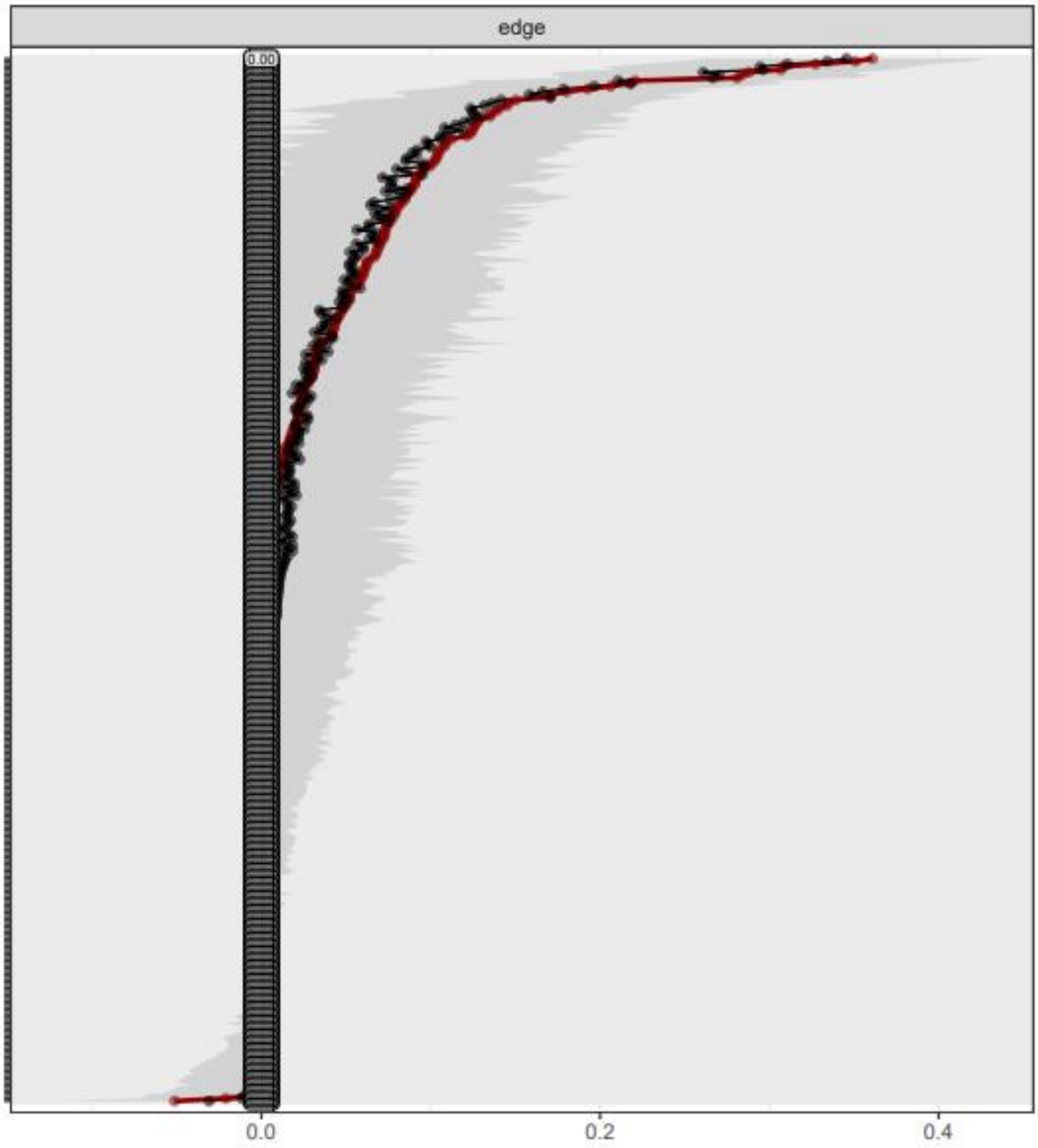

Table S15. Summary of bootstrap results for network 2b (only edges for which 95% bootstrap confidence interval did not contain zero are reported: 52 edges over 378 estimated edges). Edges are ranked by absolute edge weight. Dark blue indicate edges between ZBI and CES-D items. “prop0” indicates the proportion of times parameters were estimated to be zero.

		Bootstrap results			
Edge	Sample	Mean	CI lower	CI upper	prop0
D8--D12	0.36	0.35	0.28	0.44	0
Z5--Z9	0.35	0.33	0.26	0.44	0
D3--D6	0.33	0.31	0.24	0.41	0
D7--D20	0.31	0.30	0.22	0.39	0
Z4--Z5	0.31	0.29	0.22	0.40	0
D13--D14	0.29	0.26	0.20	0.38	0
Z10--Z17	0.28	0.27	0.19	0.37	0
D17--D18	0.28	0.27	0.19	0.38	0
Z16--Z18	0.22	0.21	0.13	0.31	0
D6--D18	0.22	0.22	0.13	0.31	0
Z18--Z19	0.21	0.20	0.12	0.30	0
D5--D20	0.19	0.18	0.11	0.27	0
Z9--Z10	0.17	0.16	0.09	0.25	0
Z15--Z16	0.18	0.17	0.09	0.27	0
Z4--Z9	0.17	0.17	0.08	0.26	0
Z7--Z19	0.15	0.14	0.06	0.23	0
Z16--Z17	0.15	0.14	0.06	0.24	0.1
Z4--Z17	0.14	0.13	0.05	0.22	0.1
D3--D18	0.13	0.13	0.05	0.22	0.1
D2--D20	0.14	0.13	0.05	0.24	0.4
Z7--Z17	0.12	0.12	0.05	0.20	0.1
D6--D20	0.13	0.11	0.05	0.21	0.1
Z17--D8	0.12	0.11	0.05	0.20	0.3
D9--D10	0.14	0.12	0.04	0.24	0.4
D5--D7	0.13	0.12	0.03	0.23	0.4
D12--D20	0.11	0.10	0.03	0.19	0.3
D4--D12	0.13	0.12	0.03	0.22	0.7
D14--D15	0.14	0.12	0.03	0.25	1.5
Z10--Z15	0.11	0.11	0.03	0.20	1
Z7--Z16	0.11	0.10	0.03	0.19	0.8
Z7--D18	0.09	0.08	0.02	0.16	1.3
D13--D20	0.10	0.09	0.02	0.18	1.8
Z5--D1	0.10	0.09	0.02	0.18	1.8
Z4--Z7	0.10	0.10	0.02	0.18	0.4
Z19--D9	0.10	0.09	0.02	0.19	1.7
D1--D3	0.10	0.09	0.02	0.19	2.1
D3--D4	0.09	0.09	0.02	0.17	1.6
D3--D15	0.13	0.11	0.02	0.23	2.5
D10--D11	0.12	0.11	0.02	0.22	2.5
D7--D11	0.11	0.10	0.02	0.20	2.1
Z10--D7	0.10	0.09	0.02	0.19	2.1
Z17--D9	0.09	0.08	0.01	0.17	3.1
Z9--D10	0.08	0.06	0.01	0.15	4.8
Z9--Z18	0.10	0.10	0.01	0.19	1.5
D12--D18	0.08	0.07	0.01	0.15	2.5
Z4--Z19	0.09	0.10	0.01	0.18	0.9
D1--D5	0.10	0.08	0.01	0.19	5.4
D6--D7	0.09	0.09	0.004	0.17	2.5
D1--D15	0.09	0.08	0.003	0.18	5.6
Z16--D12	0.08	0.07	0.003	0.15	4.5
Z4--Z18	0.09	0.09	0.002	0.17	2.5
D5--D14	0.09	0.07	0.001	0.18	5

Z4=Embarrassed of patient’s behaviour; Z5=Angry around the patient Z7=Afraid of patient’s future; Z9=Strained around the patient; Z10=Health affected by caregiving; Z15=Financially stressed; Z16=Unable to take care of the patient much; Z17=Sense of losing control over life; Z18=Wish to leave caring of the patient; Z19=Feel uncertain of what to do. D1=feeling bothered; D2=appetite changes; D3=feeling blue; D4=lack of feeling good; D5=difficulty with concentrating; D6=depressed mood; D7=everything was an effort; D8=hopelessness; D9=feeling of failure; D10=fearful; D11=sleep disturbances; D12=lack of happiness; D13=talking less; D14= lonely; D15=people unfriendly; D17=crying. D18=sadness; D20=inability to get going.

Fig. S8. Average correlations between centrality indices (strength) of networks sampled with persons dropped and the original sample (network 2b). The line indicates the means and areas indicate the range from the 2.5th to the 97.5th quantiles. Maximum drop proportions to retain correlation of 0.7 in at least 95% of the samples: 0.594.

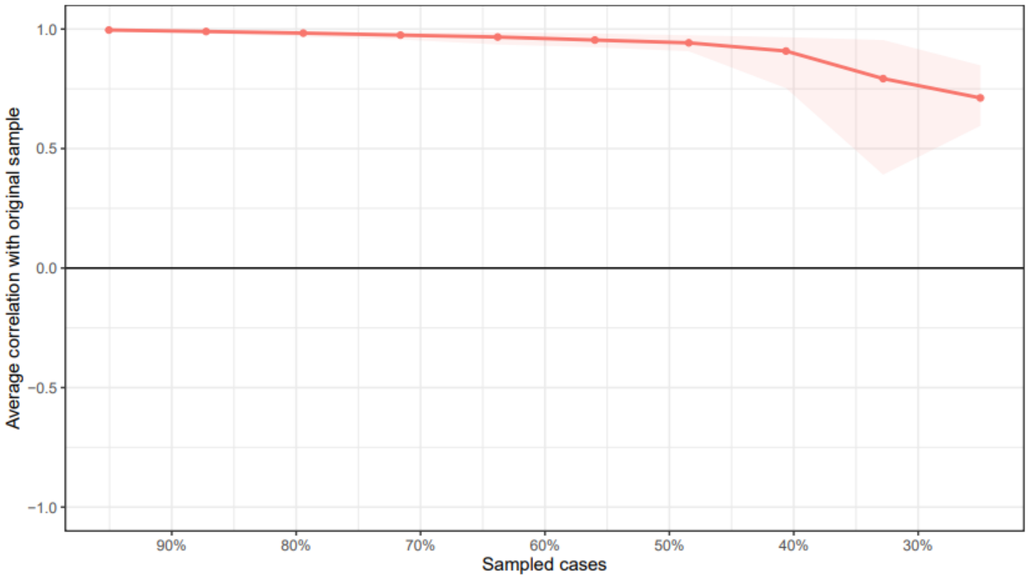

Table S16. Partial correlations matrix of network 3a (Fig. 3a): PE = Psychoeducation. Highlighted boxes indicate edges between psychoeducation and caregiving dimensions or CES-D symptoms.

	B1	B2	B3	B4	Z1	Z2	Z3	Z4	Z5	D1	D2	D3	D4	D5	D6	D7	D8	D9	D10	D11	D12	D13	D14	D15	D17	D18	D20	PE
B1																												
B2	0																											
B3	0.12	0.07																										
B4	0.02	0.11	0.29																									
Z1	0.30	0	0.10	0.06																								
Z2	0.09	0	0	0.18	0.27																							
Z3	0.05	0	0	0.09	0.11	0.18																						
Z4	0.06	0	0.02	0	0.27	0.12	0.26																					
Z5	0	0	0.10	0.01	0.15	0	0	0.004																				
D1	0.13	0	0	0	0.05	0	0.08	0	0																			
D2	0.03	0	0	0	0	0	0	0	0	0.01																		
D3	0	0	0	0	0	0	0	0	0	0.10	0.09																	
D4	0	-0.1	0	0	0	0	0	-0.01	0	0	0	0.09																
D5	0	0	0.001	0.05	0.02	0	0	0	0.03	0.09	0.03	0	0															
D6	0	0	0	0	0.03	0	0	0	0	0.04	0.01	0.32	0.04	0.03														
D7	0	0	0	0.005	0.07	0.003	0	0	0	0.04	0.04	0.01	0	0.13	0.10													
D8	0	-0.09	0	0.01	0.02	0.02	0	0	0	0	0	0.04	0.04	0.001	0.02	0												
D9	0	-0.04	0	0.003	0.11	0	0	0	0.06	0	0	0.07	0.05	0	0.04	0.01	0.05											
D10	0	0	0	0	0.04	0	0	0	0	0	0.01	0	0	0.02	0.08	0.01	0.01	0.14										
D11	0	0	0	0.04	0	0	0.03	0	0	0	0.06	0	0	0.03	0.05	0.11	0.01	0	0.12									
D12	0	-0.13	0.01	0	0.11	0.02	0	0	0.01	0.01	0.01	0	0.11	0	0	0	0.36	0.05	0	0								
D13	0	0	0	0	0	0	0	0	0	0.02	0.08	0.01	0.04	0	0	0.01	0	0.02	0.05	0.03	0.01							
D14	0	0	0.03	0.07	0.03	0.04	0.03	0	0.02	0	0.02	0.03	0.003	0.08	0.08	0.06	0	0.02	0	0.004	0.01	0.28						
D15	0	0	0.00	0.07	0	0	0.02	0	0	0.08	0	0.12	0	0	0	0.03	0	0	0.07	0	0	0.005	0.12					
D17	0	0	0.03	0	0	0	0	0	0	0	0.03	0.08	0.07	0	0.09	0	0	0.04	0	0	0	0	0.02	0				
D18	0	0	0	0.09	0.002	0	0	0	0.04	0	0	0.13	0	0	0.22	0.06	0	0.07	0.08	0.05	0.08	0	0.06	0	0.28			
D20	0	-0	0	0	0	0	0	0	0.01	0.02	0.14	0.004	0.01	0.19	0.13	0.31	0.08	0.01	0.01	0.04	0.11	0.10	0	0	0	0.08		
PE	0	0.05	0	0	-0.08	0	-0.02	-0.01	0	0	0	0	-0.05	0	0	0	-0.01	-0.01	0	0	0	0	-0.01	0	-0.04	0	0	

Table S17. Partial correlations matrix of network 3b (Fig. 3b): PE = Psychoeducation. Highlighted boxes indicate edges between psychoeducation and ZBI items from the Negative Emotion/Consequences dimension or CES-D symptoms.

	Z4	Z5	Z7	Z9	Z10	Z15	Z16	Z17	Z18	Z19	D1	D2	D3	D4	D5	D6	D7	D8	D9	D10	D11	D12	D13	D14	D15	D17	D18	D20
Z4																												
Z5	0.30																											
Z7	0.10	0																										
Z9	0.17	0.35	0																									
Z10	0	0	0	0.17																								
Z15	0	0	0.05	0	0.11																							
Z16	0	0.01	0.11	0	0.06	0.18																						
Z17	0.13	0.01	0.12	0.02	0.28	0.05	0.15																					
Z18	0.09	0.02	0	0.10	0.07	0.01	0.22	0.04																				
Z19	0.09	0.05	0.14	0.08	0.06	0	0.06	0	0.20																			
D1	0.03	0.10	0	0.03	0.003	0	0	0.03	0.06	0.02																		
D2	0	0	0	0	0.01	0	0	0	0	0	0.02																	
D3	0	0	0	0	0	0	0	0	0	0	0.10	0.09																
D4	0	0	-0.04	0	0	0	0	0	0	0	0	0	0.09															
D5	0	0	0	0.03	0.05	0.01	5E-04	0	0	0	0.09	0.03	0	0														
D6	0	0	0.02	0	0.07	0	0	0.02	0	0	0.04	0.01	0.32	0.04	0.02													
D7	0	0	0	0.02	0.10	0.04	0.02	0.01	0	0	0.04	0.04	0.01	0	0.13	0.09												
D8	0	0	0.08	0	0	0	0	0.12	0	0.01	0	0	0.03	0.05	0	0	0											
D9	0.02	0.01	0	0	0	0.06	0.002	0.09	0	0.10	0	0	0.07	0.06	0	0.03	0.005	0.03										
D10	0	0	0	0.08	0	0.002	0	0	0	0	0	0.01	0	0	0.02	0.07	0.01	0.003	0.14									
D11	0	0	0.01	0.03	0.01	0.04	0	0	0	0	0	0.06	0	0	0.03	0.05	0.11	0.004	0	0.12								
D12	0	0.01	0	0.07	0.02	0	0.08	0.02	0	0.03	0.01	0.02	0	0.12	0	0	0	0.36	0.06	0	0							
D13	0.01	0	0	0	0	0	0	0	0	0	0.02	0.08	0.01	0.04	0	0	0.01	0	0.01	0.05	0.03	0.01						
D14	0	0	0	0	0.05	0.06	0	0.07	0	0.03	0	0.02	0.03	0	0.09	0.08	0.06	0	0.01	0	0.01	0.01	0.28					
D15	0.01	0	0	0	0	0.06	0	0	0	0	0.09	0	0.12	0	0	0	0.03	0	0	0.07	0	0	0.005	0.14				
D17	0	0	0	0	0.003	0	0	0	0	0.02	0	0.03	0.08	0.07	0	0.09	0	0	0.04	0	0	0	0	0.02	0			
D18	0	0	0.09	0.02	0	0	0.01	0	0	0	0	0	0.14	0	0	0.22	0.06	0	0.07	0.07	0.05	0.08	0	0.07	0	0.28		
D20	0	0	0	0	0	0	0.02	0	-0	0	0.02	0.14	0.005	0.01	0.19	0.13	0.30	0.07	0.01	0.01	0.04	0.11	0.10	0	0	0	0.08	
PE	0	-0.03	0	0	0	-0.05	-0.04	-0.06	-0.01	-0.03	0	0	0	-0.06	0	0	0	-0.001	-0.01	0	0	0	0	-0.01	0	-0.04	0	0
